# Himalayan *Saccharomyces eubayanus* Genome Sequences Reveal Genetic Markers Explaining Heterotic Maltotriose Consumption by Saccharomyces pastorianus Hybrids

**DOI:** 10.1128/AEM.01516-19

**Published:** 2019-10-30

**Authors:** Nick Brouwers, Anja Brickwedde, Arthur R. Gorter de Vries, Marcel van den Broek, Susan M. Weening, Lieke van den Eijnden, Jasper A. Diderich, Feng-Yan Bai, Jack T. Pronk, Jean-Marc G. Daran

**Affiliations:** a Department of Biotechnology, Delft University of Technology, Delft, The Netherlands; b State Key Laboratory of Mycology, Institute of Microbiology, Chinese Academy of Sciences, Beijing, China; Rutgers, The State University of New Jersey

**Keywords:** *Saccharomyces eubayanus*, α-oligoglucoside metabolism, heterosis, domestication, experimental evolution sequencing, brewing, hybridization

## Abstract

Saccharomyces pastorianus strains are hybrids of Saccharomyces cerevisiae and Saccharomyces eubayanus that have been domesticated for centuries in lager beer brewing environments. As sequences and structures of S. pastorianus genomes are being resolved, molecular mechanisms and evolutionary origins of several industrially relevant phenotypes remain unknown. This study investigates how maltotriose metabolism, a key feature in brewing, may have arisen in early S. eubayanus × S. cerevisiae hybrids. To address this question, we generated a nearly complete genome assembly of Himalayan S. eubayanus strains of the Holarctic subclade. This group of strains has been proposed to be the S. eubayanus subgenome origin of current S. pastorianus strains. The Himalayan S. eubayanus genomes harbored several copies of an S. eubayanus *AGT1* (*SeAGT1*) α-oligoglucoside transporter gene with high sequence identity to genes encountered in S. pastorianus. Although Himalayan S. eubayanus strains cannot grow on maltose and maltotriose, their maltose-hydrolase and *SeMALT1* and *SeAGT1* maltose transporter genes complemented the corresponding null mutants of S. cerevisiae. Expression, in Himalayan S. eubayanus of a functional S. cerevisiae maltose metabolism regulator gene (*MALx3*) enabled growth on oligoglucosides. The hypothesis that the maltotriose-positive phenotype in S. pastorianus is a result of heterosis was experimentally tested by constructing an S. cerevisiae × S. eubayanus laboratory hybrid with a complement of maltose metabolism genes that resembles that of current S. pastorianus strains. The ability of this hybrid to consume maltotriose in brewer’s wort demonstrated regulatory cross talk between subgenomes and thereby validated this hypothesis. These results support experimentally the new postulated hypothesis on the evolutionary origin of an essential phenotype of lager brewing strains and valuable knowledge for industrial exploitation of laboratory-made S. pastorianus-like hybrids.

**IMPORTANCE** S. pastorianus, an S. cerevisiae × S. eubayanus hybrid, is used for production of lager beer, the most produced alcoholic beverage worldwide. It emerged by spontaneous hybridization and colonized early lager brewing processes. Despite accumulation and analysis of genome sequencing data of S. pastorianus parental genomes, the genetic blueprint of industrially relevant phenotypes remains unresolved. Assimilation of maltotriose, an abundant sugar in wort, has been postulated to be inherited from the S. cerevisiae parent. Here, we demonstrate that although Asian S. eubayanus isolates harbor a functional maltotriose transporter *SeAGT1* gene, they are unable to grow on α-oligoglucosides, but expression of S. cerevisiae regulator *MAL13* (*ScMAL13*) was sufficient to restore growth on trisaccharides. We hypothesized that the S. pastorianus maltotriose phenotype results from regulatory interaction between S. cerevisiae maltose transcription activator and the promoter of *SeAGT1*. We experimentally confirmed the heterotic nature of the phenotype, and thus these results provide experimental evidence of the evolutionary origin of an essential phenotype of lager brewing strains.

## Abstract (precis)

S. pastorianus, an S. cerevisiae × S. eubayanus hybrid, is used for production of lager beer, the most produced alcoholic beverage worldwide. It emerged by spontaneous hybridization and colonized early lager brewing processes. Despite accumulation and analysis of genome sequencing data of S. pastorianus parental genomes, the genetic blueprint of industrially relevant phenotypes remains unresolved. Assimilation of maltotriose, an abundant sugar in wort, has been postulated to be inherited from the S. cerevisiae parent. Here, we demonstrate that although Asian S. eubayanus isolates harbor a functional maltotriose transporter *SeAGT1* gene, they are unable to grow on α-oligoglucosides, but expression of S. cerevisiae regulator *MAL13* (*ScMAL13*) was sufficient to restore growth on trisaccharides. We hypothesized that the S. pastorianus maltotriose phenotype results from regulatory interaction between S. cerevisiae maltose transcription activator and the promoter of *SeAGT1*. We experimentally confirmed the heterotic nature of the phenotype, and thus these results provide experimental evidence of the evolutionary origin of an essential phenotype of lager brewing strains.

## INTRODUCTION

*Saccharomyces pastorianus* is an interspecific hybrid of Saccharomyces cerevisiae and Saccharomyces eubayanus (1–4). S. pastorianus strains are widely used for production of lager beer, which is currently the most produced alcoholic beverage worldwide. Lager brewing requires alcoholic fermentation at relatively low temperatures. S. pastorianus was hypothesized to have emerged by spontaneous hybridization and to have colonized early lager brewing processes due to a combination of cold tolerance inherited from S. eubayanus and superior fermentation kinetics inherited from S. cerevisiae (5–7). Lager beer is brewed from barley wort, whose sugar composition consists, by weight, of approximately 15% glucose, 60% maltose, and 25% maltotriose (8). During wort fermentation, maltotriose is generally utilized only after glucose and maltose are depleted, while its consumption is also relatively slow and often incomplete (9–11).

Complete sugar utilization is desirable for lager beer fermentation to optimize concentrations of ethanol and flavor compounds and to avoid residual sweetness (12). While S. pastorianus and S. cerevisiae strains are capable of consuming maltotriose, none of the wild isolates of S. eubayanus characterized thus far have been shown to possess this trait (6, 13, 14). These observations led to the hypothesis that the ability of S. pastorianus to ferment maltotriose was inherited from S. cerevisiae (5–7, 13, 15).

The genetic information for maltose utilization is well conserved in *Saccharomyces* species and depends on three gene families. *MALT* genes encode plasma membrane proton symporters with various substrate specificities and affinities (16, 17); *MALS* genes encode α-glucosidases that hydrolyze α-oligoglucosides into glucose, while *MALR* genes encode a regulator required for transcriptional induction of *MALT* and *MALS* genes by maltose (18, 19). In *Saccharomyces* species, maltose utilization genes are generally organized in *MAL* loci. These loci contain a *MALT* gene (called *ScMALx1* and *SeMALTx* in S. cerevisiae and S. eubayanus, respectively), a *MALS* gene referred to as *ScMALx2* or *SeMALSx*, and an *MALR* gene referred to as *ScMALx3* or *SeMALRx* (13, 20). In the absence of glucose and presence of maltose, the MalR regulator binds a bidirectional promoter, thereby simultaneously activating expression of *MALT* and *MALS* genes (21).

The *ScMAL1*-*ScMAL4* and *ScMAL6* loci of S. cerevisiae as well as the *SeMAL1-SeMAL4* loci of S. eubayanus are located in subtelomeric regions (13, 22–24). While all S. cerevisiae *Sc*Malx1 transporters transport maltose, only *Sc*Mal11 is able to also transport maltotriose (9). *ScMAL11* (also known as *ScAGT1*) shares only 57% nucleotide identity with other *ScMALx1* genes (25). The four *SeMALT* (*SeMALT1* to *SeMALT4*) genes identified in the genome of the Patagonian type strain FM1318/CBS 12357 of S. eubayanus were shown to encode functional maltose transporters, but none of these genes enabled maltotriose transport (13). While no clear *ScAGT1* ortholog was found in S. eubayanus CBS 12357^T^, such an ortholog was recently found in the genomes of two North American isolates assigned to the Holarctic subclade of S. eubayanus (14).

S. pastorianus inherited *MAL* genes from both S. cerevisiae and S. eubayanus (2, 4, 26). However, the S. cerevisiae-derived maltotriose transporter gene *ScAGT1* is truncated and, therefore, nonfunctional in S. pastorianus (10). Instead, maltotriose consumption by S. pastorianus strains was attributed to *SeAGT1* and S. pastorianus *MTY1* and *MTT1* genes (27–30). In S. pastorianus, *SeAGT1* is located on S. eubayanus chromosome XV (ChrXV) and was, therefore, already before the identification of an *AGT1* ortholog in Holarctic S. eubayanus strains (14), assumed to originate from S. eubayanus (2). *SpMTY1*, also referred to as *SpMTT1*, is located on S. cerevisiae ChrVII and has less than 92% sequence identity with other *Saccharomyces* maltose transporters (28). However, *SpMTY1* contains sequence patches with high similarity to maltose transporters from S. eubayanus and Saccharomyces paradoxus (32). Recently, two independent laboratory evolution studies with S. eubayanus demonstrated that recombination of different *SeMALT* genes yielded chimeric, neo-functionalized genes that encoded maltotriose transporters (14, 32). *SpMTY1* may have resulted from successive introgressions of maltose transporter genes from S. cerevisiae, S. eubayanus, and S. paradoxus.

Recently made S. cerevisiae × S. eubayanus laboratory hybrids showed lager brewing performance similar to that of S. pastorianus strains, also with respect to maltotriose utilization (5, 6, 15, 33). In these hybrids, maltotriose consumption depended on the presence of a functional *Sc*Agt1 transporter encoded by the S. cerevisiae subgenome (34). However, in view of the nonfunctionality of *ScAGT1* in current S. pastorianus strains, these laboratory hybrids did not fully recapitulate the genetic landscape of S. pastorianus with respect to maltotriose fermentation (2, 6, 33).

Studies on laboratory hybrids based on S. eubayanus strains whose genomes are more closely related to the S. eubayanus subgenome of S. pastorianus strains than that of the Patagonian type strain CBS 12357 might generate new insights into the evolution of maltotriose utilization in S. pastorianus. To date, Himalayan S. eubayanus isolates show the highest sequence identity with the S. eubayanus subgenome of S. pastorianus, with up to 99.82% identity, in contrast to 99.56% for S. eubayanus CBS 12357^T^ (35).

Here, we investigated if and how the genomes of Himalayan S. eubayanus strains could have contributed to maltotriose utilization in the earliest hybrid ancestors of current S. pastorianus strains. To this end, we generated chromosome-level genome assemblies of these strains by long-read DNA sequencing. Since the Himalayan strains were unable to utilize maltotriose, we functionally characterized the assembled *MAL* genes and identified genetic determinants that prevented maltotriose utilization. Subsequently, a laboratory hybrid of a representative Himalayan S. eubayanus strain with a maltotriose-deficient ale strain of S. cerevisiae was generated to investigate the genetics of maltotriose utilization in a hybrid context. We discuss the implications of the experimental results for the proposed role and origin of *SeAGT1* in S. pastorianus and for the potential of hybridization to enable maltotriose consumption in novel *Saccharomyces* hybrids.

## RESULTS

### Sequencing of Himalayan S. eubayanus strains revealed variations of subtelomeric regions and the presence of novel putative maltose transporter genes.

It has been proposed that the S. eubayanus genetic pool of S. pastorianus was inherited from an ancestor of the Asian S. eubayanus lineage (35). With 99.82% identity, the Himalayan S. eubayanus strains CDFM21L.1 and ABFM5L.1 that belong to the Holarctic lineage (36) present the closest characterized relatives of the S. eubayanus ancestor of lager brewing yeasts. However, this distance was based on a limited sequencing space (35), and the analysis did not investigate the presence of specific S. eubayanus genetic markers found in S. pastorianus hybrids. Therefore, we sequenced the genome of the Himalayan S. eubayanus strain CDFM21L.1 with a combination of long-read and short-read techniques (Oxford Nanopore MinION and Illumina technologies, respectively) to generate a nearly complete draft reference genome sequence. The resulting CDFM21L.1 genome assembly comprised 19 contigs, including the mitochondrial genome. All chromosomes were completely assembled from telomere to telomere, except for chromosome XII, which was fragmented into 3 contigs due to the repetitive ribosomal DNA (rDNA) region and manually assembled into a single scaffold. With a total size of 12,034,875 bp, this assembly represents the first nearly complete draft genome of an S. eubayanus strain of the Holarctic clade (36).

**FIG 1:**
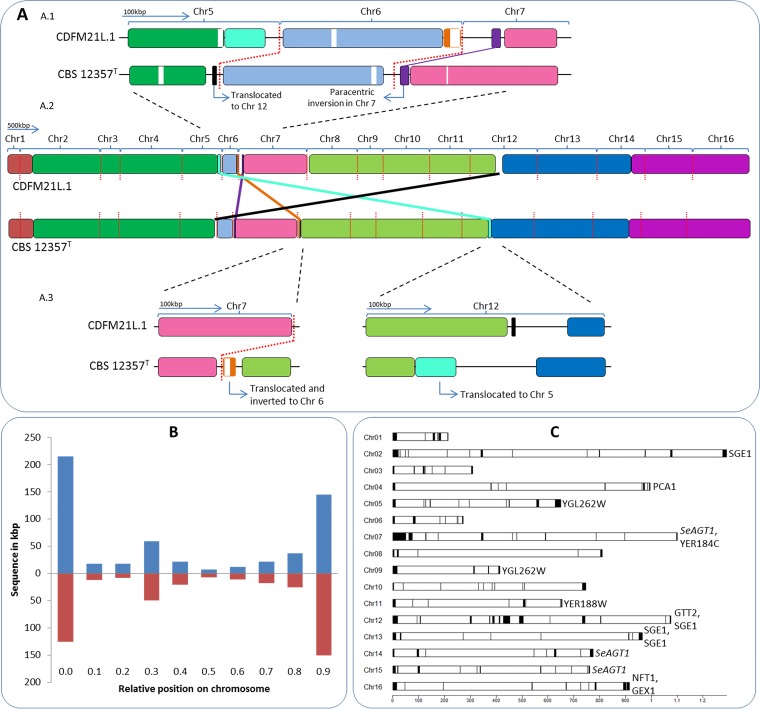
Genome comparison between CDFM21L.1 and CBS 12357^T^. (A) Translocations in CDFM21L.1 relative to CBS 12357^T^. The diagram in the first panel shows a magnification of the right end of ChrV, the entire ChrVI, and the left end of ChrVII, and it displays the paracentric inversion of the subtelomeric region of ChrVII left arm (approximately 8 kbp). The second panel represents the concatenated whole-genome alignment of S. eubayanus strains CBS 12357 and CDFM21L.1. The red vertical lines indicate the chromosome separations. The third panel shows, on the left, a magnification of a translocation of approximately 12 kbp from the left subtelomeric region of chromosome VIII to the right subtelomeric region of chromosome VI and a reciprocal translocation between approximately 20 kbp from the right subtelomeric region of chromosome V and approximately 60 kbp from the center of the chromosome. Genome synteny is indicated with colored blocks. (B) Relative chromosome position of gene presence differences between CDFM21L.1 (blue) and CBS 12357^T^ (red). (C) Representation of the assembled CDFM21L.1 S. eubayanus chromosomes. The black boxes denote newly added sequences. New annotated open reading frames and gene entries modified relative to the CBS 12357^T^ draft genome are shown (13).

Chromosome-level assemblies were hitherto only available for the Patagonia B-clade strain CBS 12357^T^ (1, 13). We identified three major structural differences in CDFM21L.1 relative to the structure of CBS 12357^T^ using Mauve (37): (i) a paracentric inversion in the subtelomeric region of chromosome VII involving approximately 8 kbp, (ii) a translocation of approximately 12 kbp from the left subtelomeric region of chromosome VIII to the right subtelomeric region of chromosome VI, and (iii) a reciprocal translocation between approximately 20 kbp from the right subtelomeric region of chromosome V and approximately 60 kbp from the center of chromosome XII (Fig. 1A). All structural variation involved subtelomeric regions, in accordance with their known relative instability (38–40).

An alignment comparison of the CDFM21L.1 and CBS 12357^T^ genomes with MUMmer revealed that 557 kb were unique to CDFM21L.1, and, reciprocally, 428 kb were unique to CBS 12357^T^. Sequences unique to CBS 12357^T^ (3.6% of its genome) and to CDFM21L.1 (4.6% of its genome) were located primarily in subtelomeric regions and in repetitive regions, such as rDNA on chromosome XII (Fig. 1B). Out of the 32 subtelomeric regions, 23 exhibited absence of synteny. Conserved synteny was observed for subtelomeric regions on ChrIII (left), ChrIV (left and right), ChrVI (left), ChrIX (right), ChrXI (right), ChrXII (right), ChrXIV (left), and ChrXV (right) (see File S1 and Table S1 in the supplemental material).

The 428 kb of sequence that was absent in the Himalayan S. eubayanus strain included 99 annotated open reading frames (ORFs) (File S1). Of the 99 ORFs that were (partly) affected, 11 were completely absent in CDFM21L.1, involving mostly genes implicated in iron transport facilitation (File S1). The 557 kb of sequence that was not present in CBS 12357^T^ included 113 annotated ORFs (File S1). Of these 113 ORFs, 15 were completely absent in CBS 12357^T^. These 15 ORFs showed an overrepresentation of genes involved in transmembrane transport (Fisher’s exact test, *P* = 4.8E−5) (Fig. 1C).

**FIG 2:**
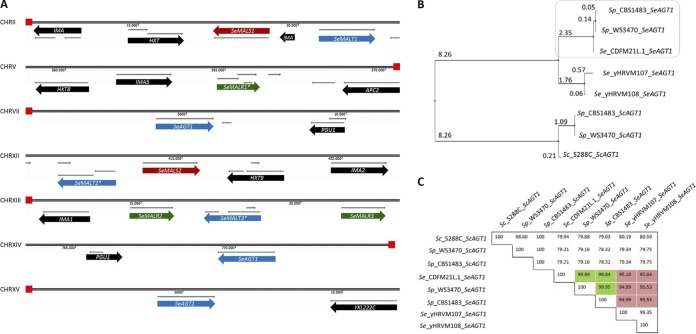
Organization of subtelomeric regions involving *MAL* genes and *SeAGT1* in CDFM21L.1. (A) Chromosome sections are represented as lines, and red boxes denote telomeres. The CDFM21L.1 genome harbors three *SeMALT* genes in which *SeMALT2* and *SeMALT3* have a mutation resulting in an early stop codon and truncated protein (denoted with *). Three copies of *SeAGT1* were found close to the telomeres on chromosomes VII, XIV, and XV. Furthermore, there are two intact *SeMALS* genes on ChrII and ChrXII and three *SeMALR* genes on ChrV and ChrXIII whose copy on ChrV is also mutated (SeMALR1*). The gene and interval sizes are approximately to scale. Transporter genes *SeAGT1*, *SeMALT1*, *SeMALT2*, and *SeMALT3* are denoted with blue arrows, the hydrolase genes *SeMALS1* and *SeMALS2* are denoted with red arrows, and the regulator genes *SeMALR1*, *SeMALR2*, and *SeMALR3* are denoted with green arrows. Any other genes are shown with black arrows. (B) Phylogeny of *Saccharomyces SeAGT1* genes described in S. cerevisiae, S. eubayanus, and the lager brewing hybrid S. pastorianus. (C) Nucleotide percentage identities between *AGT1* orthologs from S. cerevisiae, S. eubayanus, and the lager brewing hybrid S. pastorianus. Green indicates highest similarity between *SeAGT1* and *SeAGT1* genes from S. pastorianus strains CBS 1483 and WS3470. Red indicates similarity between *SeAGT1* from North American strains with *SeAGT1* genes from Asian S. eubayanus and S. pastorianus strains CBS 1483 and WS3470.

Of the 15 ORFs unique to CDFM21L.1, three were identical orthologs of S. cerevisiae *MAL11* (*AGT1*) (Table S1). These three ORFs were found in the subtelomeric regions of chromosomes VII, XIV, and XV. Their sequence similarity values with the S. cerevisiae CEN.PK113.7D and S. pastorianus CBS 1483 *MAL11* and *AGT1* genes were 82.7% and 99.89%, respectively. In addition to these *SeAGT1* genes, the CDFM21L.1 genome sequence harbored genes encoding three maltose transporters (*SeMALTx*), two maltases (*SeMALSx*), and three regulators (*SeMALRx*). In contrast to the situation in S. eubayanus CBS 12357^T^, none of the *SeMAL* genes formed a canonical *MAL* locus in CDFM21L.1 (Fig. 2). A systematic sequence inspection of these CDFM21L.1 *SeMAL* genes revealed mutations that prematurely interrupted the reading frames of *SeMALR1*^ChrV^ (_706_TGA_708_), *SeMALT2*^ChrXII^ (_694_TGA_696_), and *SeMALT*3^ChrXIII^ (_1045_TAA_1047_).

In addition to the S. eubayanus CDFM21L.1 strain, a second Himalayan S. eubayanus isolate (ABFM5L.1) was sequenced. These two strains were 99.97% genetically identical at the nucleotide level, their *MAL* genes were syntenic, and the premature stop codons in *SeMALR1* (ChrV), *SeMALT2* (ChrXII), and *SeMALT*3 (ChrXIII) were conserved. Two additional mutations were identified in one of the three *SeAGT1* genes. A nucleotide variation at positions 53 and 939 (T instead of an A and A instead of a G) resulted in a glycine-to-valine and arginine-to-lysine change, respectively.

### Paradoxically, Himalayan S. eubayanus strains do not utilize maltose and maltotriose.

**FIG 3:**
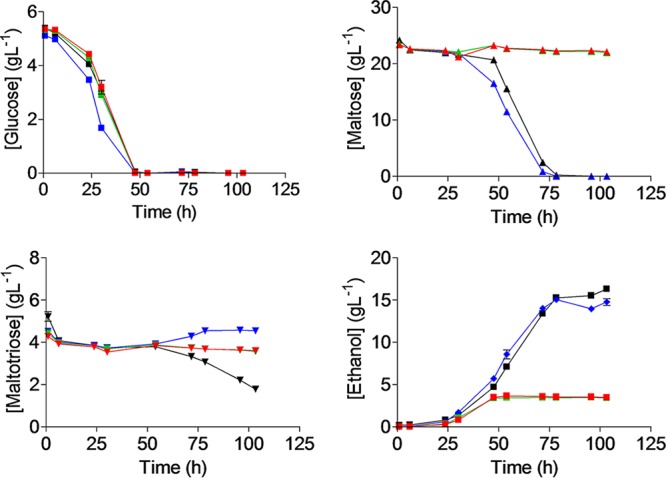
Characterization of sugar consumption of S. pastorianus CBS 1483 (black) and S. eubayanus CBS 12357^T^ (blue), CDFM21L.1 (red), and ABFM5L.1 (green) on wort. For every sample, glucose (▪), maltose (▲), maltotriose (▼), and ethanol (⧫) were measured from the supernatant. Strains were grown at 12°C for 110 h in infusion Neubor flasks. Samples were filtered through a 0.22-μm-pore-size filter and analyzed by HPLC. Data represent averages and standard deviations of three biological replicates.

Identification of *SeAGT1* in the two Himalayan S. eubayanus strains suggests an ability to grow not only on maltose but also on maltotriose. Strains from the Holarctic clade have previously been hypothesized to be the donor of the S. eubayanus subgenome in S. pastorianus hybrids (35, 36). However, no physiological data regarding their ability to grow on the sugars present in wort are available. To assess their growth characteristics, the Asian S. eubayanus strains CDFM21L.1 and ABFM5L.1, the Patagonian S. eubayanus type strain CBS 12357^T^, and the S. pastorianus strain CBS 1483 were grown on diluted industrial brewer’s wort at 12°C. As reported previously, S. pastorianus strain CBS 1483 could utilize all three sugars but did not fully consume maltotriose (Fig. 3) (11). Also in accordance with previous observations (6), CBS 12357^T^ consumed glucose and maltose completely but left maltotriose untouched. However, in marked contrast to S. eubayanus CBS 12357^T^, neither CDFM21L.1 nor ABFM5L.1 consumed maltose after growth on glucose. Moreover, like CBS 12357^T^, maltotriose was not metabolized by these two S. eubayanus strains. While in CBS 12357^T^ an ability to grow on maltose and an inability to grow on maltotriose could be readily attributed to its *MAL* gene complement, CDFM21L.1 and ABFM5L.1 failed to grow on maltose even though they appeared to contain complete genes encoding maltose (*SeMALT1* and *SeAGT1*) and maltotriose (*SeAGT1*) transporters.

### Growth defects on maltose and maltotriose are caused by deficiency of the regulatory *Se*MalR proteins in S. eubayanus CDFM21L.1.

**FIG 4:**
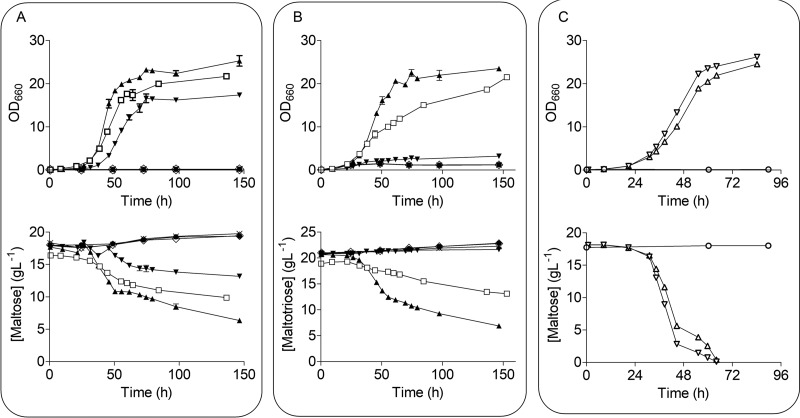
Overexpression of *SeMALT*, *SeAGT1*, and *SeMALS* genes in a maltose-negative background S. cerevisiae strain. Maltose-negative background strain IMZ616 (X), IMX1365 overexpressing *ScMAL11* (▼), IMX1702 overexpressing *SeMALT1* (⧫), IMX1704 overexpressing *SeMALT2* (↓), IMX1706 overexpressing *SeMALT3* (●), and IMX1708 overexpressing *SeAGT1* (☐) were grown on SM with 2% maltose or maltotriose at 20°C. It is worth mentioning that the symbols corresponding to the strains IMZ616, IMX1704, and IMX1708 are highly overlapping and therefore might be difficult to visualize. Growth on maltose (A) and on maltotriose (B) was monitored based on optical density (OD_660_), and concentrations of maltose and maltotriose in culture supernatants were measured by HPLC. Data are presented as averages and standard deviations of two biological replicates. (C) IMX1313 overexpressing only *ScMAL31* (○), IMZ752 overexpressing *ScMAL31* and *SeMALS1* (▵), and IMZ753 overexpressing *ScMAL31* and *SeMALS2* (∇) grown on SM with 2% maltose. Growth was monitored based on optical density measurement at 660 nm (OD_660_), and maltose in culture supernatants was measured by HPLC. Data represent averages and standard deviations of two biological replicates.

The recent characterization of maltose metabolism in CBS 12357^T^ showed that the coding regions of transcriptionally silent maltose transporter genes in S. eubayanus can potentially encode functional proteins (13). The inability of the Himalayan S. eubayanus isolates to grow on α-oligosaccharides precluded direct testing of transporter gene functionality by deletion studies. Instead, these genes were expressed in S. cerevisiae IMZ616, which is devoid of all native maltose metabolism genes (41). The CDFM21L.1 transporter gene *SeMALT1*, *SeMALT2*, *SeMALT3*, or *SeAGT1* was integrated at the *ScSGA1* locus in IMZ616 along with the S. cerevisiae maltase gene *ScMAL12* (13), yielding a series of strains overexpressing a single transporter [(IMX1702 (*SeMALT1*), IMX1704 (*SeMALT2*), IMX1706 (*SeMALT3*), and IMX1708 (*SeAGT1*)]. These strains, as well as the negative- and positive-control strains IMZ616 and IMX1365 (IMZ616 expressing *ScAGT1* and *ScMAL12*), were grown on synthetic medium (SM) supplemented with either maltose (SMM) or maltotriose (SMMt). On maltose, not only the positive-control strain IMX1365 but also IMX1702 (*SeMALT1*) and IMX1708 (*SeAGT1*) were able to grow on maltose, consuming 30 and 60%, respectively, of the initially present maltose after 100 h (Fig. 4A). As anticipated, the *SeMALT2* and *SeMALT3* alleles with premature stop codons did not support growth on maltose. Of the two strains that grew on maltose, only IMX1708 (*SeAGT1*) also grew on maltotriose. These results demonstrate that *SeAGT1* from a Holarctic S. eubayanus encoded a functional maltotriose transporter and, consequently, that the inability of Holarctic strains to grow on maltose and maltotriose was not caused by transporter dysfunctionality. In addition to transport, metabolism of α-oligoglucosides requires maltase activity. Functionality of the putative *SeMALS1* and *SeMALS2* maltase genes was tested by constitutive expression in strain IMZ616, together with a functional *ScMAL31* transporter gene, yielding strains IMZ752 and IMZ753, respectively. The maltase-negative strain IMX1313 was used as negative control. In SM with maltose, both IMZ752 (*SeMALS1*) and IMZ753 (*SeMALS2*) grew and completely consumed maltose within 65 h, demonstrating functionality of both hydrolase genes (Fig. 4C).

**FIG 5:**
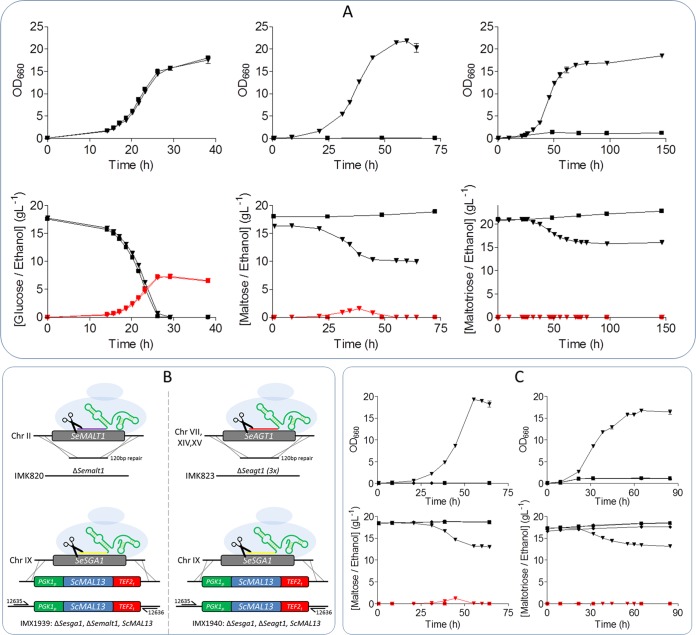
Integration of *ScMAL13* in CDFM21L.1 restores growth on maltose and maltotriose and enables native *SeMALT1* and *SeAGT1* characterization in knockout strains IMK820 and IMK823. (A) Characterization of S. eubayanus IMX1765 overexpressing *ScMAL13* (▼) and CDFM21L.1 (▪) on SM with glucose, maltose, or maltotriose at 20°C. The OD_660_ was measured (black), and sugar (black) and ethanol (red) concentrations were determined from the supernatant by HPLC. (B) Overview of constructed knockout strains. Knockouts of *SeMALT1* (IMK820) and *SeAGT1* (IMK823) were made with CRISPR-Cas9. Subsequently the *SeSGA1* locus was replaced by *ScPGK1_p_-ScMAL13-ScTEF2_t_* using CRISPR-Cas9 in both strains, resulting in IMX1939 and IMX1940, respectively. (C) S. eubayanus strains IMK820 (▪), IMK823 (▲), IMX1939 (▼), and IMX1940 (⧫) were characterized on SM with maltose or maltotriose at 20°C. The OD_660_ was measured (black), and sugar (black) and ethanol (red) concentrations were determined from the supernatant by HPLC. It is worth mentioning that the symbols corresponding to the strains IMK820, IMK823, and IMX1940 are highly overlapping and therefore might be difficult to visualize. All data represent averages and standard deviations of biological duplicates.

In S. cerevisiae transcriptional regulation of *MALx2* and *MALx1* genes is tightly controlled by a transcription factor encoded by *MALx3* genes. Malx3 binds an activating site located in the bidirectional promoters that control expression of *MALx2* and *MALx1* genes (42, 43). To test whether absence of maltose consumption in Himalayan S. eubayanus strains was caused by a lack of transcriptional upregulation of *SeMALT* and *SeMALS*, the S. cerevisiae *ScMAL13* gene was integrated at the *SeSGA1* locus in S. eubayanus CDFM21L.1, under the control of a constitutive *ScPGK1* promoter and *ScTEF2* terminator. *ScMAL13* expression in CDFM21L.1 enabled growth on maltose and maltotriose (Fig. 5A), indicating that a lack of transcriptional upregulation was indeed the cause of the parental strain’s inability to grow on these oligoglucosides. However, consumption of maltose and maltotriose was incomplete, and consumed sugars were almost exclusively respired, as no ethanol was measured after 60 h of cultivation.

The possibility to grow an engineered variant of S. eubayanus CDFM21L.1 on α-oligoglucosides offered an opportunity to study transporter function in its native context. Complementary functional characterization by gene deletion of *SeMALT1* and *SeAGT1* was performed using CRISPR-Cas9 genome editing method (13, 44). Deletion of *SeMALT1* and *SeAGT1* in CDFM21L.1 resulted in strains IMK820 and IMK823, respectively. Complete deletion of *SeAGT1* required disruption of six alleles. To confirm the complete removal of all copies, the genome of IMK823 was sequenced. Mapping reads onto the reference S. eubayanus CDFM21L.1 genome assembly confirmed that all six alleles were removed simultaneously. Subsequently, the regulator expression cassette (*ScPGK1_p_-ScMAL13-ScTEF2_t_*) was integrated in IMK820 and IMK823 at the *SeSGA1* locus, yielding strains IMX1939 and IMX1940, respectively (Fig. 5B). The four deletion strains IMK820 (*SemalT1*Δ), IMK823 (*Seagt1*Δ), IMX1939 (*SemalT1*Δ *Sesga1*Δ::*ScMAL13*), and IMX1940 (*Seagt1*Δ *Sesga1*Δ::*ScMAL13*) were characterized on SM with glucose (SMG), SMM, or SMMt. All four strains were able to grow on glucose (Fig. S1). While strains IMK820, IMK823 and IMX1940 were unable to grow on maltose or maltotriose (Fig. 5C), strain IMX1939 (*SemalT1*Δ *Sesga1*Δ::*SeMAL13*), which harbored functional *SeAGT1* copies, grew on maltose as well as on maltotriose. However, after 64 h of growth, these sugars were only partially consumed. Only 1.2 g liter^−1^ ethanol was produced from maltose, and no ethanol formation was observed during growth on maltotriose. The low ethanol concentration and the relatively high optical density at 600 nm (OD_660_) suggest that, under the experimental conditions, strain IMX1939 exhibited a Crabtree-negative phenotype and exclusively respired maltotriose. S. eubayanus IMX1940 (*Seagt1*Δ *Sesga1*Δ::*SeMAL13*) did not consume maltotriose after 84 h of incubation. Moreover, despite the presence of *SeMALT1*, which encoded a functional maltose transporter upon expression in S. cerevisiae IMZ616, strain IMX1940 was also unable to consume maltose.

**FIG 6:**
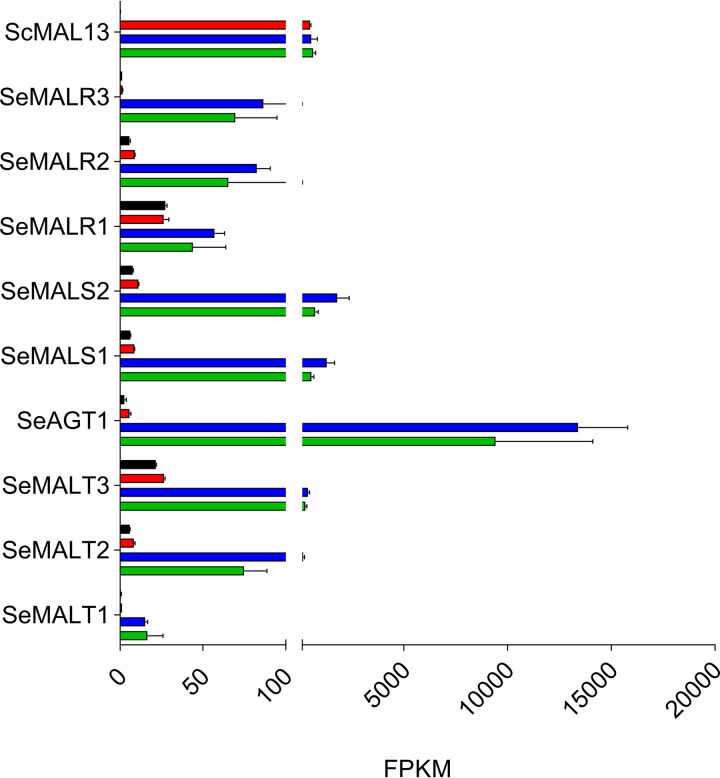
Expression levels of maltose metabolism genes in CDFM21L.1 and IMX1765. Normalized transcript levels of maltose metabolism genes from CDFM21L.1 mid-exponential phase grown on glucose (black) and from IMX1765 mid-exponential phase grown on glucose (red), maltose (blue), and maltotriose (green) at 20°C were calculated from duplicate RNA sequencing experiments (2 × 150 bp) using the FPKM method. All data represent averages and standard deviations of biological duplicates.

In addition to a functional Malx3 transcription factor, transcriptional activation of *MAL* genes also requires presence of a *cis*-regulatory motif in the promoter of regulated genes. Transcriptome analysis of S. eubayanus CBS 12357^T^ recently showed that absence of a canonical *cis*-regulatory motif in *SeMALT1* and *SeMALT3* of S. eubayanus CBS 12357^T^ caused a deficiency in their expression (13). To further explore regulation of *SeMAL* and *SeAGT1* genes, we investigated the impact of carbon sources on the genome-wide transcriptome and, specifically, on transcriptional activation of genes involved in maltose metabolism. Duplicate cultures of S. eubayanus strain IMX1765 (*ScPGK1_p_-ScMAL13-ScTEF2_t_*) were grown on SMG, SMM, and SMMt at 20°C and sampled in mid-exponential phase. After mRNA isolation and processing, sequencing reads were mapped onto the newly annotated S. eubayanus CDFM21L.1 genome to calculate the number of fragments per kilobase per million reads mapped for the gene of interest (FPKM). The heterologous regulator *ScMAL13*, expressed from the constitutive *ScPGK1* promoter (*ScPGK1_p_*) displayed the same expression levels in glucose- and maltose-grown cultures. Although *ScMAL13* was efficiently expressed on glucose, none of the nine S. eubayanus maltose genes (the three identical *SeAGT1* copies being undistinguishable) were transcriptionally induced under these conditions (Fig. 6 and Table S2), confirming that the hierarchical regulatory role of glucose catabolite repression (42, 45) also takes place in S. eubayanus. During growth on maltose, all nine genes were significantly upregulated relative to levels in glucose-grown cultures, but large variations in expression levels were observed. The maltase genes *SeMALS1* and *SeMALS2* and the transporter gene *SeAGT1* showed the highest upregulation, with fold changes of 148, 161, and 2,355 respectively. Although upregulated *SeMALT1* displayed a fold change of 13, its normalized expression in maltose-grown cultures was 886-fold lower than that of *SeAGT1*. This weaker upregulation might explain why, despite the ability of its coding region to support synthesis of a functional maltose transporter, *SeMALT1* alone could not restore growth on maltose. The transcriptome data also revealed that the absence of maltose induction in CDFM21L.1 was not associated with defective *cis*-regulatory elements in *SeMALR* promoter sequences since the regulator genes were properly activated; instead, these results would suggest that the *Se*MalR regulators are not functional.

### Hybridization of two maltotriose-deficient S. eubayanus and S. cerevisiae lineages results in heterosis through regulatory cross talk.

The genetic makeup of S. pastorianus lager brewing yeasts strongly indicates that they originate from hybridization of S. cerevisiae and S. eubayanus parental lineages that were both unable to metabolize maltotriose (2). This hypothesis is consistent with the recurrent mutation in the S. cerevisiae *AGT1* allele of S. pastorianus strains as well as with the inability of Himalayan strains of S. eubayanus to grow on these oligoglucosides.

**FIG 7:**
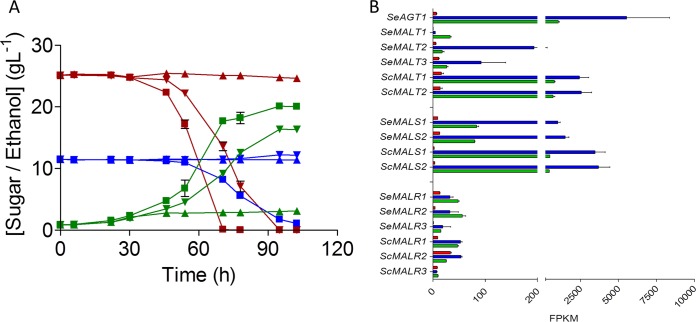
Hybridization of maltotriose-deficient S. cerevisiae and S. eubayanus strains leading to cross talk restoring maltotriose utilization explains the S. pastorianus phenotype. (A) Characterization of S. cerevisiae CBC-1 (▼), S. eubayanus CDFM21L.1 (▲), and hybrid HTSH020 (▪) on mock wort at 12°C. Consumption of maltose (red) and maltotriose (blue) and production of ethanol (green) were measured from the supernatant by HPLC. Data represent averages and standard deviations from biological triplicates. (B) Normalized transcript levels of maltose metabolism genes from HTSH020 mid-exponential phase grown on glucose (red), maltose (blue), and maltotriose (green) at 20°C were calculated from duplicate RNA sequencing experiments (2 by 150 bp) using the FPKM method. All data represent averages and standard deviations of two biological duplicates.

Spores of the Himalayan S. eubayanus CDFM21L.1 were hybridized with S. cerevisiae CBC-1. This top-fermenting S. cerevisiae is recommended for cask and bottle conditioning and unable to consume maltotriose (Lallemand, Montreal, Canada). Analysis of the CBC-1 assembly, obtained by a combination of long- and short-read sequencing, linked its maltotriose-negative phenotype to a total absence of the *MAL11* (*AGT1*) gene. The resulting laboratory interspecific hybrid HTSH020 was characterized at 12°C on synthetic wort, a defined medium whose composition resembles that of brewer’s wort. While S. eubayanus CDFM21L.1 consumed only glucose and S. cerevisiae CBC-1 consumed glucose and maltose after 103 h (Fig. 7A), the interspecific hybrid HTSH020 completely consumed glucose and maltose and partially consumed maltotriose after 105 h, thus resembling characteristics of S. pastorianus strains (e.g., CBS 1483) (11). In addition to this gain of function, the hybrid HTSH020 outperformed both of its parents in maltose consumption since it depleted this sugar in 70 h instead of the 95 h for strain CBC-1. Since S. cerevisiae grows generally more slowly at 12°C, the experiments were also performed at 20°C, at which temperature HTSH020 consumed all maltose 16 h earlier than CBC-1 (Fig. S2).

Transcriptome analysis of the hybrid strain HTSH020 grown on SM with different carbon sources showed that *SeAGT1* expression was repressed during growth on glucose, with a normalized expression level of 7 FPKM (Fig. 7B). When grown on SM with maltose, *SeAGT1*, *SeMALS1*, and *SeMALS2* were significantly induced, with fold increases of 816, 109, and 116, respectively (Fig. 7B and Table S3). Although *SeMALT1* and *SeMALT2* were induced, these transporters do not contribute to maltose metabolism due to truncation of their ORFs. These transcriptome data demonstrated that *SeAGT1* and *SeMALS* genes are induced by regulatory cross talk between regulators encoded from the CBC-1 S. cerevisiae subgenome and maltotriose transporter genes harbored by the S. eubayanus genome. This laboratory hybridization experiment may be the closest reproduction yet of how, centuries ago, maltotriose fermentation capacity arose in the first hybrid ancestor of S. pastorianus.

## DISCUSSION

The ability to consume maltose and maltotriose represents a key performance indicator of S. pastorianus lager brewing strains (10). This study demonstrates how mating of S. cerevisiae and S. eubayanus strains that cannot themselves ferment maltotriose can yield maltotriose-fermenting hybrids. This laboratory study illustrates how, centuries ago, maltotriose fermentation capacity may have arisen in the first hybrid ancestor of S. pastorianus.

While the origin of the S. eubayanus parent of S. pastorianus strains is still under debate (46–48), phylogenetic analysis suggested a Far East Asian origin (35). However, this interpretation was based on a limited sequencing space and was constrained by the quality of available sequence assemblies. Since an ortholog of *SeAGT1* had previously been found only in the S. eubayanus subgenome of S. pastorianus strains, this finding revived the discussion on the geographical origin of the ancestral S. eubayanus parent (14). The high-quality, annotated genome assemblies of the Himalayan S. eubayanus strains CDFM21L.1 and ABFM5L.1 presented in the present study revealed several copies of *SeAGT1*, whose very high sequence identity with S. pastorianus *SeAGT1* is consistent with the previously proposed Asian origin of the S. eubayanus subgenome of S. pastorianus (14, 35, 36). Next, genome sequence comparison of the Patagonian B subclade S. eubayanus strain CBS 12357^T^ and the Holarctic subclade strain CDFM21L.1 revealed homoplasy of *SeAGT1*, probably reflecting that these subclades evolved in different ecological niches. The variation in genes encoding iron transport facilitators between the Patagonian and Himalayan S. eubayanus lineages further supports this idea of a difference of the natural habitat of these yeasts. The abundance of these transporters in CBS 12357^T^ could indicate a lower iron concentration in the environment of Patagonian S. eubayanus, therefore requiring a higher transport capacity to sustain sufficient intake. It could also indicate the presence of other organisms competing for this essential trace element in its ecosystem.

The S. eubayanus wild stock whose genome sequence most closely corresponds to the S. eubayanus subgenome of S. pastorianus originates from the Tibetan plateau of the Himalayas (35). However, the first S. cerevisiae × S. eubayanus hybrid, from which current lager yeasts evolved by centuries of domestication, likely originates from a region between Bavaria and Bohemia in Central Europe. So far, European S. eubayanus isolates have not been reported. This may indicate that the original hybridization event occurred elsewhere or that the ancestral European lineage became extinct. The recent detection, in a metagenomics analysis of samples from the Italian Alps, of internal transcribed spacer 1 (ITS1) sequences corresponding to S. eubayanus could indicate that a wild European lineage exists after all (49).

Functional characterization by heterologous complementation of an S. cerevisiae mutant strain established that the *Se*Agt1 transporters from the Himalayan S. eubayanus strains CDFM21L.1 and ABFM5L supported uptake of maltose and maltotriose. After it was shown that these strains also encoded a functional maltase gene, their inability to grow on maltose and maltotriose was attributed to an inability to transcriptionally upregulate maltose metabolism genes likely caused by regulator loss of function. In S. cerevisiae and, to some extent, in S. eubayanus strains of the Patagonian B subclade such as CBS 12357^T^ (13, 24, 50), MAL loci exhibit a specific organization in which a transporter (*MALT*) and a hydrolase (*MALS*) gene are expressed from the same bidirectional promoter and are located adjacent to a regulator gene (*MALR*) (19). In contrast, of the seven genomic regions harboring *MAL* genes in the two Asian S. eubayanus strains, none showed this canonical organization (Fig. 2), and the subtelomeric regions carrying *SeAGT1* did not harbor sequences similar to hydrolase or regulator genes. Subtelomeric regions harboring the other *MAL* genes indicated intensive reorganization as a result of recombination. In particular, subtelomeric regions on ChrII, ChrV, and ChrXII provide clear indications for recombination events that scattered genes from ancestral MAL1 and MAL2 loci over several chromosomes. A similar interpretation could explain the reorganization MAL3 on ChrXIII (Fig. 2). Similar events may have contributed to loss of function of the MAL regulators (MalR), as exemplified by the occurrence of a nonsynonymous mutation in S*eMALR1* resulting in loss of function. These rearrangements did not, however, inactivate the *cis*-regulatory sequences of the *MAL* genes since complementation with a functional *ScMAL13* allele caused induction of most *SeMAL* genes (Fig. 6 and 7B) and, thereby, the heterotic maltotriose-positive phenotype of the hybrid strain HTSH020. Together with the high copy number of *SeAGT1*, this heterotic complementation may have been the main driver for colonization of low-temperature brewing processes by the early hybrid ancestors of current S. pastorianus strains.

Recent work on adaptation to brewing environments of laboratory S. cerevisiae × S. eubayanus hybrids showed loss of maltotriose utilization during serial transfer in wort (34). A similar loss of maltotriose utilization is frequently encountered in S. cerevisiae ale strains (52), as well as in some Saaz-type S. pastorianus strains (53). This is thus in contrast with retention of a maltotriose assimilation phenotype by Frohberg-type S. pastorianus strains. This may have been facilitated by the occurrence of multiple copies of the *SeAGT1* gene in the S. eubayanus ancestor, which could act as a sequence buffer to counteracting adverse effects of gene copy loss. The recent release of the first long-read sequencing assembly of S. pastorianus enabled a precise chromosomal mapping of the maltose metabolism genes (54) and showed that the Frohberg-type S. pastorianus strain CBS 1483 harbored one copy of *SeAGT1* on the S. eubayanus ChrXV section (as in CDFM21L.1) of the chimeric chromosome formed from S. eubayanus ChrXV (*Se*ChrXV) and *Se*ChrVIII (54).

Differential retention and loss of maltotriose consumption in S. pastorianus lineages may reflect different brewing process conditions during domestication. In modern brewing processes based on high-gravity wort, cell division is largely constrained to the glucose and maltose phases, which occur before depletion of nitrogen sources (55). It may be envisaged that, in early lager brewing processes, nonstandardized mashing processes generated wort with a higher maltotriose content, which would have allowed for continued yeast growth during the maltotriose consumption phase. During serial transfer on sugar mixtures, the selective advantage of consuming a specific sugar from a mixture correlates with the number of generations on that sugar during each cycle (56, 57). Such conditions would therefore have conferred a significant selective advantage to a maltotriose-assimilating S. cerevisiae × S. eubayanus hybrid, especially if, similar to current ale yeasts, the S. cerevisiae parent was unable to ferment maltotriose.

The heterotic phenotype that was reconstructed in the interspecies S. cerevisiae × S. eubayanus hybrid HTSH020 resulted from combination of dominant and recessive genetic variations from both parental genomes. S. eubayanus contributed the *SeAGT1* gene and its functional *cis*-regulatory sequences but also harbored recessive mutations in *MALR* genes that allowed full expression of the heterotic phenotype. These mutations were complemented with a set of S. cerevisiae genes including a functional *MALR* and an absence of the *ScAGT1* gene to match the mutations found in S. pastorianus (2). Although some S. pastorianus strains harbor an additional maltotriose transporter encoded by *SpMTT1* (28), this gene was recently proposed to have emerged after the original hybridization event as a result of repeated recombination between *MALT* genes from both subgenomes (32). It is worthwhile mentioning that this hypothesis would also stand if the parental S. cerevisiae was carrying a functional *AGT1* gene, as do about 60% of S. cerevisiae (ale) strains (52). The gene could have been mutagenized and lost its function through domestication (34).

Maltotriose fermentation is likely not the only heterotic phenotype of S. pastorianus strains. Flocculation and formation of complex aroma profiles (26, 58) are phenotypes that are not fully understood and difficult to reproduce and also might result from heterosis (34).

Laboratory-made S. cerevisiae × S. eubayanus hybrids hold great potential for brewing process intensification and for increasing product diversity. In addition to increasing our understanding of the evolutionary history of lager yeast genomes, this study has implications for the design of new hybrids. Hitherto, laboratory crosses of S. cerevisiae × S. eubayanus strains were designed based on a combination of dominant traits of the parental strains. Our results show that recessive traits can be just as important as contributors to the genetic diversity of such hybrids.

## MATERIALS AND METHODS

### Strains and maintenance.

All strains used in this study are listed in Table 1. Stock cultures of S. eubayanus and S. cerevisiae strains were grown in YPD medium (10 g liter^−1^ yeast extract, 20 g liter^−1^ peptone, and 20 g liter^−1^ glucose) until late exponential phase, complemented with sterile glycerol to a final concentration of 30% (vol/vol) and stored at –80°C as 1-ml aliquots until further use.

**TABLE 1:** *Saccharomyces* strains used in this study

Name	Species	Relevant genotype^*a*^	Source or reference
CDFM21L.1	S. eubayanus	Wild type, Mal^−^ Mtt^−^	35
ABFM5L.1	S. eubayanus	Wild type, Mal^−^ Mtt^−^	35
CBS 12357	S. eubayanus	Wild type, Mal^+^ Mtt^−^	1; Westerdijk Institute^*c*^
CBS 1483	S. pastorianus	Wild type, Mal^+^ Mtt^+^	Westerdijk Institute
CEN.PK113-7D	S. cerevisiae	*MAT***a** *MAL1x MAL2x MAL3x MAL4x MAL2-8C SUC2 LEU2 URA3*	84
IMZ616	S. cerevisiae	*MAT***a** *ura3-52 LEU2 MAL2-8C malΔ mp2/3hΔ suc2Δ ima1Δ ima2*Δ *ima3Δ ima4Δ ima5Δ* pUDC156 (*Spcas9 URA3 ARS4 CEN6*)	41
IMX1365	S. cerevisiae	*MAT***a** *ura3-52 LEU2 MAL2-8C malΔ mph2/3Δ suc2Δ ima1Δ ima2Δ ima3Δ ima4Δ ima5Δ* pUDC156 (*URA3 cas9*) *sga1Δ*::*ScTDH3_p_-ScMAL12-ScADH1_t_ ScTEF1_p-_ScAGT1-ScCYC1_t_*	13
IMX1702	S. cerevisiae	*MAT***a** *ura3-52 LEU2 MAL2-8C malΔ mph2/3Δ suc2Δ ima1Δ ima2Δ ima3Δ ima4Δ ima5Δ* pUDC156 (*URA3 cas9*) *sga1Δ*::*ScTDH3_p_-ScMAL12-ScADH1_t_ ScTEF1_p_-SeMALT1-ScCYC1_t_*	This study
IMX1704	S. cerevisiae	*MAT***a** *ura3-52 LEU2 MAL2-8C malΔ mph2/3Δ suc2Δ ima1Δ ima2Δ ima3Δ ima4Δ ima5Δ* pUDC156 (*URA3 cas9*) *sga1Δ*::*ScTDH3_p_-ScMAL12-ScADH1_t_ ScTEF1_p_-SeMALT2-ScCYC1_t_*	This study
IMX1706	S. cerevisiae	*MAT***a** *ura3-52 LEU2 MAL2-8C malΔ mph2/3Δ suc2Δ ima1Δ ima2Δ ima3Δ ima4Δ ima5Δ* pUDC156 (*URA3 cas9*) *sga1Δ*::*ScTDH3_p_-ScMAL12-ScADH1_t_ ScTEF1_p_-SeMALT3-ScCYC1_t_*	This study
IMX 1708	S. cerevisiae	*MAT***a** *ura3-52 LEU2 MAL2-8C malΔ mph2/3Δ suc2Δ ima1Δ ima2Δ ima3Δ ima4Δ ima5Δ* pUDC156 (*URA3 cas9*) *sga1Δ*::*ScTDH3_p_-ScMAL12-ScADH1_t_ ScTEF1_p_-SeAGT1-ScCYC1_t_*	This study
IMX1313	S. cerevisiae	*MAT***a** *ura3-52 LEU2 MAL2-8C malΔ mph2/3Δ suc2Δ ima1Δ ima2Δ ima3Δ ima4Δ ima5Δ* sga1*Δ*::*ScTEF1_p_-ScMAL31-ScCYC1_t_* pUDC156 (*URA3 cas9*)	This study
IMX1313Δ	S. cerevisiae	*MAT***a** *ura3-52 LEU2 MAL2-8C malΔ mph2/3Δ suc2Δ ima1Δ ima2Δ ima3Δ ima4Δ ima5Δ* sga1*Δ*::*ScTEF1_p_-ScMAL31-ScCYC1_t_*	This study
IMZ752	S. cerevisiae	*MAT***a** *ura3-52 LEU2 MAL2-8C malΔ mph2/3Δ suc2Δ ima1Δ ima2Δ ima3Δ ima4Δ ima5Δ* sga1*Δ*::*ScTEF1_p_-ScMAL31-ScCYC1_t_* pUDE843 (*ori* [ColE1] *bla* 2μ *ScTDH3_p_-SeMALS1-ScADH1_t_ URA3*)	This study
IMZ753	S. cerevisiae	*MAT***a** *ura3-52 LEU2 MAL2-8C malΔ mph2/3Δ suc2Δ ima1Δ ima2Δ ima3Δ ima4Δ ima5Δ* sga1*Δ*::*ScTEF1_p_-ScMAL31-ScCYC1_t_* pUDE844 (*ori* [ColE1] *bla* 2μ *ScTDH3_p_-SeMALS2-ScADH1_t_ URA3*)	This study
IMK820^*b*^	S. eubayanus	*MAT***a***/MATα Semalt1Δ*/*Semalt1Δ*	This study
IMK823^*b*^	S. eubayanus	*MAT***a***/MATα Seagt1Δ*/*Seagt1Δ* (X3)	This study
IMX1939^*b*^	S. eubayanus	*MAT***a***/MATα Semalt1Δ*/*Semalt1Δ Sesga1Δ*::*ScMAL13*/*Sesga1Δ*::*ScMAL1*3	This study
IMX1940^*b*^	S. eubayanus	*MAT***a***/MATα Seagt1*Δ/*Seagt1*Δ *Sesga1Δ*::*ScMAL13*/*Sesga1Δ*::*ScMAL13*	This study
IMX1762^*b*^	S. eubayanus	*MAT***a***/MATα Sesga1Δ*::*ScMAL12*/*Sesga1Δ*::*ScMAL12*	This study
IMX1765^*b*^	S. eubayanus	*MAT***a***/MATα Sesga1Δ*::*ScMAL13*/*Sesga1Δ*::*ScMAL13*	This study
CBC-1	S. cerevisiae	*MAT***a***/MATα* Mal^+^ Mtt^−^	Lallemand
HTSH020	S. cerevisiae × S. eubayanus	*MAT***a***/MATα* Mal^+^ Mtt^+^	This study

a The abbreviation *malΔ* indicates *mal11-mal12*::*loxP mal21-mal22*::*loxP mal31-32*::*loxP*. Mal and Mtt denote the maltose and maltotriose phenotype, respectively.

b Direct derivatives of the wild-type S. eubayanus strain CDFM21L.1.

*^c^*Westerdijk Fungal Biodiversity Institute (www.westerdijkinstitute.nl/).

### Media and cultivation.

S. eubayanus batch cultures were grown on synthetic medium (SM) containing 3.0 g liter^−1^ KH_2_PO_4_, 5.0 g liter^−1^ (NH_4_)_2_SO_4_, 0.5 g liter^−1^ MgSO_4_·7H_2_O, 1 ml liter^−1^ trace element solution, and 1 ml liter^−1^ vitamin solution (59). The pH was set to 6.0 with 2 M KOH prior to autoclaving at 120°C for 20 min. Vitamin solutions were sterilized by filtration and added to the sterile medium. Concentrated sugar solutions were autoclaved at 110°C for 20 min or filter sterilized and added to the sterile flasks to give a final concentration of 20 g liter^−1^ glucose (SMG), maltose (SMM), or maltotriose (SMMt). With the exception of IMZ752 and IMZ753, S. cerevisiae batch cultures were grown on SM supplemented with 150 mg liter^−1^ uracil (60) to compensate for loss of plasmid pUDC156 (Table 2) that carried the *Spcas9* endonuclease gene and supplemented with 20 g liter^−1^ glucose (SM_u_G), maltose (SM_u_M), or maltotriose (SM_u_Mt). All batch cultures were grown in 250-ml shake flasks with a working volume of 50 ml. The cultures were inoculated at an initial OD_660_ of 0.1 and incubated under an air atmosphere and shaken at 200 rpm and at 20°C in a New Brunswick Innova 44 incubator (Eppendorf Nederland B.V., Nijmegen, The Netherlands).

**TABLE 2:** Plasmids used in this study

Plasmid	Relevant genotype	Source or reference
p426-TEF-amdS	*ori* (ColE1) *bla* 2μ amdSYM *TEF1_p_-CYC1_t_*	62
pUD444	*ori* (ColE1) *bla* 2μ amdSYM *ScTEF1_p_-ScMAL31-ScCYC1_t_*	This study
pUD794	*ori* (ColE1) *bla* 2μ amdSYM *ScTEF1_p_-SeMALT1-ScCYC1_t_*	This study
pUD795	*ori* (ColE1) *bla* 2μ amdSYM *ScTEF1_p_-SeMALT2-ScCYC1_t_*	This study
pUD796	*ori* (ColE1) *bla* 2μ amdSYM *ScTEF1_p_-SeMALT3-ScCYC1_t_*	This study
pUD797	*ori* (ColE1) *bla* 2μ amdSYM *ScTEF1_p_-SeAGT1-ScCYC1_t_*	This study
pUDE044	*ori* (ColE1) *bla* 2μ *ScTDH3_p_-ScMAL12-ScADH1_t_ URA3*	63
pUDE843	*ori* (ColE1) *bla* 2μ *ScTDH3_p_-SeMALS1-ScADH1_t_ URA3*	This study
pUDE844	*ori* (ColE1) *bla* 2μ *ScTDH3_p_-SeMALS2-ScADH1_t_ URA3*	This study
pUDE780	*ori* (ColE1) *bla* 2μ *ScPGK1_p_-ScMAL13-ScTEF2_t_ URA3*	This study
pUDP002	*ori* (ColE1) *bla* panARSopt *Hyg ScTDH3_p_*-BsaI-BsaI-*ScCYC1_t_ AaTEF1_p_-Spcas9*^D147Y P411T^-*ScPHO5_t_*	66
pUDP004	*ori* (ColE1) *bla* panARSopt amdSYM *ScTDH3_p_*-BsaI-BsaI-Sc*CYC1_t_ AaTEF1_p_-Spcas9*^D147Y P411T^-*ScPHO5_t_*	44
pUDP052	*ori* (ColE1) *bla* panARSopt amdSYM *ScTDH3_p_*-gRNA*_SGA1_-ScCYC1_t_ AaTEF1_p_-Sp*cas9^D147Y P411T^-*ScPHO5_t_*	13
pUDP091	*ori* (ColE1) *bla* panARSopt amdSYM *ScTDH3_p_*-gRNA*_SeMALT1_-ScCYC1_t_ AaTEF1_p_-Sp*cas9^D147Y P411T^-*ScPHO5_t_*	This study
pUDP090	*ori* (ColE1) *bla* panARSopt amdSYM *ScTDH3_p_*-gRNA*_SeAGT1_-ScCYC1_t_ AaTEF1_p_-Sp*cas9^D147Y P411T^-*ScPHO5_t_*	This study
pUDR119	*ori* (ColE1) *bla* 2μ amdSYM *SNR5*2*_p_*_-_gRNA*_ScSGA1_-SUP4_t_*	68
pYTK074	*ori* (ColE1) *cat URA3*	64
pYTK082	*cat* 2μ	64
pYTK083	*ori* (ColE1) *bla*	64
pUD631	*ori* (ColE1) *bla* gRNA*_SeMALT1_*	13
pUD634	*ori* (ColE1) *bla* gRNA*_SeAGT1_*	This study
pUDC156	*ori* (ColE1) *bla ARS4 CEN6 URA3 Spcas9*	41

S. eubayanus strains transformed with plasmid pUDP052 carrying a guide RNA targeting *SeSGA1* [(gRNA*_SeSGA1_*)], pUDP091 (gRNA*_SeMALT1_*), and pUDP090 (gRNA*_SeAGT1_*) were selected on modified SMG medium in which (NH_4_)_2_SO_4_ was replaced by 6.6 g liter^−1^ K_2_SO_4_ and 10 mM acetamide (SM_Ace_G) (61). SM-based solid medium contained 2% Bacto agar (BD Biosciences, Franklin Lakes, NJ). S. cerevisiae strains expressing either *SeMALT*, *SeMALS*, or *ScMALR* were selected on SM_Ace_G. For plasmid propagation, Escherichia coli XL1 Blue-derived strains (Agilent Technologies, Santa Clara, CA) were grown in lysogeny broth (LB) medium (10 g liter^−1^ tryptone, 5 g liter^−1^ yeast extract, 5 g liter^−1^ NaCl) supplied with 100 mg liter^−1^ ampicillin. Synthetic wort medium (SWM) for growth studies contained 14.4 g liter^−1^ glucose, 2.3 g liter^−1^ fructose, 85.9 g liter^−1^ maltose, 26.8 g liter^−1^ maltotriose, 5 g liter^−1^ (NH_4_)_2_SO_4_, 3 g liter^−1^ KH_2_PO_4_, 0.5 g liter^−1^ MgSO_4_·7H_2_O, 1 ml liter^−1^ trace element solution, and 1 ml liter^−1^ vitamin solution, supplemented with the anaerobic growth factors ergosterol and Tween 80 (0.01 g liter^−1^ and 0.42 g liter^−1^ respectively), as previously described (59).

Industrial wort (containing 14.4 g liter^−1^ glucose, 85.9 g liter^−1^ maltose, 26.8 g liter^−1^ maltotriose, 2.3 g liter^−1^ fructose, and 269 mg liter^−1^ free amino nitrogen [FAN]) was provided by Heineken Supply Chain B.V. (Zoeterwoude, The Netherlands). The wort was supplemented with 1.5 mg liter^−1^ of Zn^2+^ by addition of ZnSO_4_·7H_2_O, autoclaved for 30 min at 121°C, filtered using Nalgene 0.2-μm-pore size surfactant-free cellulose acetate (SFCA) bottle-top filters (Thermo Scientific), and diluted with sterile demineralized water. Sporulation medium consisted of 2% (wt/vol) potassium acetate (KAc) in MilliQ water set to pH 7.0 with KOH, autoclaved at 121°C for 20 min.

### Microaerobic growth experiments.

Microaerobic cultures were grown in 250-ml airlock-capped Neubor infusion bottles (38-mm neck; Dijkstra, Lelystad, The Netherlands) containing 200 ml of 3-fold-diluted industrial wort supplemented with 0.4 ml liter^−1^ pluronic antifoam (Sigma-Aldrich, St. Louis, MO). Bottle caps were equipped with a 0.5- by 16-mm Microlance needle (BD Biosciences) and sealed with cotton to prevent pressure buildup. Sampling was performed aseptically with 3.5-ml syringes using an 0.8- by 50-mm Microlance needle (BD Biosciences). Microaerobic cultures were inoculated at an OD_660_ of 0.1 from stationary-phase precultures in 50 ml of Bio-One Cellstar Cellreactor tubes (Sigma-Aldrich) containing 30 ml of the same medium and grown for 4 days at 12°C. Bottles were incubated at 12°C and shaken at 200 rpm in a New Brunswick Innova 43/43R shaker (Eppendorf Nederland B.V.). At regular intervals, 3.5-ml samples were collected in deep 24-well plates (EnzyScreen BV, Heemstede, The Netherlands) using a LiHa liquid handler (Tecan, Männedorf, Switzerland) to measure the OD_660_ and external metabolites. Thirty microliters of each sample was diluted 5-fold in demineralized water in a 96-well plate, and the OD_660_ was measured with a Magellan Infinite 200 Pro spectrophotometer (Tecan). From the remaining sample, 150 μl was vacuum filter sterilized using 0.2-μm-pore-size multiscreen filter plates (Merck, Darmstadt, Germany) for high-pressure liquid chromatography (HPLC) measurements.

### Analytical methods.

Optical densities of yeast cultures were measured with a Libra S11 spectrophotometer (Biochrom, Cambridge, United Kingdom) at a wavelength of 660 nm. Biomass dry weight was measured by filtering 10-ml culture samples over preweighed nitrocellulose filters with a pore size of 0.45 μm. Filters were washed with 10 ml of water, dried in a microwave oven (20 min at 350 W), and reweighed. Sugars were measured using an Agilent Infinity 1260 series high-pressure liquid chromatograph (Agilent Technologies) using a Bio-Rad Aminex HPX-87H column at 65°C with 5 mM sulfuric acid at a flow rate of 0.8 ml min^−1^. Compounds were measured using a refractive index detector (RID) at 35°C. Samples were centrifuged at 13,000 × *g* for 5 min to collect supernatant or filter sterilized with a 0.2-μm-pore-size filter before analysis.

### Plasmid construction.

Plasmids used and constructed in this study are listed in Table 2, and oligonucleotide primers used in this study are listed in Table 3. Coding regions of *SeMALT1*, *SeMALT2*, *SeMALT3*, and *SeAGT1* were amplified from CDFM21L.1 genomic DNA with Phusion High-Fidelity DNA polymerase (Thermo Scientific), according to the supplier’s instructions, with the primers pairs 12355/12356, 12357/12358, 12359/12360, and 12361/12362, respectively. The coding sequence of *ScMAL31* was amplified from CEN.PK113-7D genomic DNA with Phusion High-Fidelity DNA polymerase (Thermo Scientific), according to the supplier’s instructions, with the primer pair 9942/9943. Each primer carried a 40-bp extension complementary to the plasmid backbone of p426-TEF-amdS (62), which was PCR amplified using Phusion High-Fidelity DNA polymerase (Thermo Scientific) and the primer pair 7812/5921. Each transporter fragment was assembled with the p426-TEF-amdS backbone fragment using NEBuilder HiFi DNA Assembly (New England Biolabs, Ipswich, MA), resulting in plasmids pUD444 (*ScMAL31*), pUD794 (*SeMALT1*), pUD795 (*SeMALT2*), pUD796 (*SeMALT3*), and pUD797 (*SeAGT1*). All plasmids were verified for correct assembly by Sanger sequencing (Baseclear, Leiden, The Netherlands).

**TABLE 3:** Primers used in this study

Primer	Sequence (5′–3′)	Purpose
12355	GCTCATTAGAAAGAAAGCATAGCAATCTAATCTAAGTTTTGAGACCATCAGTTAACAATG	Amplification of *SeMALT1*
12356	GGAGGGCGTGAATGTAAGCGTGACATAACTAATTACATGATTATTGATTCGCGACTGACGC	Amplification of *SeMALT1*
12357	GCTCATTAGAAAGAAAGCATAGCAATCTAATCTAAGTTTTGCTATTAGGCAACTATGAAGGG	Amplification of *SeMALT2*
12358	GGAGGGCGTGAATGTAAGCGTGACATAACTAATTACATGACACTAAGAGTCATCAAATCATGAG	Amplification of *SeMALT2*
12359	GCTCATTAGAAAGAAAGCATAGCAATCTAATCTAAGTTTTCAAATGAGATCGAGAACGGC	Amplification of *SeMALT3*
12360	GGAGGGCGTGAATGTAAGCGTGACATAACTAATTACATGAGCCATAATTGTTTATTGAATAAGAGTC	Amplification of *SeMALT3*
12361	GCTCATTAGAAAGAAAGCATAGCAATCTAATCTAAGTTTTCGTCCTCTGCAAGAGTGTAT	Amplification of *SeAGT1*
12362	GGAGGGCGTGAATGTAAGCGTGACATAACTAATTACATGACCACTTAAATATGCTCACGG	Amplification of *SeAGT1*
14451	AGTTTCGACGGATTCTAGAACTAGTCATAAATGACTATTTCTTTTGCGCATCCAG	Amplification of SeMAL12_chr2
14452	AGTTTCGACGGATTCTAGAACTAGTCATAAATGACTATTTCTTCTGAACACCCGG	Amplification of SeMAL12_chr12
14453	GCCAACCCTCGAGGTCGACGGTATCGATAATTACTTGGCATAGTACAATCTACCTTCC	Amplification of *SeMAL12*
9942	GCTCATTAGAAAGAAAGCATAGCAATCTAATCTAAGTTTTCGGCTGTGTACATTTCATCCTGAGTGAGCGCATATTGCATAAG	Amplification of *ScMAL31*
9943	GGAGGGCGTGAATGTAAGCGTGACATAACTAATTACATGACGCCGTATCTACCTACTGGCTAAAAAAATC	Amplification of *ScMAL31*
5921	AAAACTTAGATTAGATTGCTATGCTTTCTTTCTAATGAGC	Amplification of p426-TEF-amdS backbone
7812	TCATGTAATTAGTTATGTCACGCTTACATTC	Amplification of p426-TEF-amdS backbone
14449	TTATCGATACCGTCGACCTC	Amplification of pUDE044 backbone
14450	TTATGACTAGTTCTAGAATCCGTCG	Amplification of pUDE044 backbone
9421	AAGCATCGTCTCATCGGTCTCAAACGTATTTTAGATTCCTGACTTCAACTC	Amplification of Sc-pPKG1
9422	TTATGCCGTCTCAGGTCTCACATATGTTTTATATTTGTTGTAAAAAGTAGATAATTAC	Amplification of Sc-pPKG1
10884	AAGCATCGTCTCATCGGTCTCAATCCGAGTAATAATTATTGCTTCCATATAATATTTTTATATAC	Amplification of Sc-TEF2t
10885	TTATGCCGTCTCAGGTCTCACAGCAGGAAACGTAAATTACAAGGTATATAC	Amplification of Sc-TEF2t
12915	TGAGCCACCCGGTCTCATATGACTTTAACTAAGCAAACATGCG	Amplification of *ScMAL13*
12916	GGTAGTCGGGGGTCTCAGGATTCAAGGGTCTATGTCTTCATTATCC	Amplification of *ScMAL13*
9036	TTTACAATATAGTGATAATCGTGGACTAGAGCAAGATTTCAAATAAGTAACAGCAGCAAACATAGCTTCAAAATGTTTCTACTCCTTTTTTAC	Integration in *ScSGA1*
9039	CACCTTTCGAGAGGACGATGCCCGTGTCTAAATGATTCGACCAGCCTAAGAATGTTCAACGCCGCAAATTAAAGCCTTCG	Integration in *ScSGA1* with maltase
11018	TGTAAATATCTAGGAAATACACTTGTGTATACTTCTCGCTTTTCTTTTATTTTTTTTTGTGCCGCAAATTAAAGCCTTCG	Integration in *ScSGA1* without maltase
11320	ATGAAAAATATACTTTCGCTGGTAGGAAGAAAGGAAAATACCCCAGAAGATGTGACGGCGCGTCCGCAAGTTGATAACATTATTGACCGGTTCTCAAGCGCGAGTCAACAGGCGTTATGA	Repair fragment AS2.4948 Δ*AGT1* (+)
11321	TCATAACGCCTGTTGACTCGCGCTTGAGAACCGGTCAATAATGTTATCAACTTGCGGACGCGCCGTCACATCTTCTGGGGTATTTTCCTTTCTTCCTACCAGCGAAAGTATATTTTTCAT	Repair fragment AS2.4948 Δ*AGT1* (−)
12442	ATGAAAGGTCTATCTTCAATATTGAATAGAAAGAGAAACGAAAGTGATTCGATTTCCAGTAGTGGATCCGTTTCTCATAAAAACAGACTCTGGTGATATCACACATGAAGACCTAAAGTA	Repair fragment AS2.4948 Δ*MALT1* (+)
12443	TACTTTAGGTCTTCATGTGTGATATCACCAGAGTCTGTTTTTATGAGAAACGGATCCACTACTGGAAATCGAATCACTTTCGTTTCTCTTTCTATTCAATATTGAAGATAGACCTTTCAT	Repair fragment AS2.4948 Δ*MALT1* (−)
11671	AGGTTCCTGGGCAGTGAAGC	Diagnostic out-out^*a*^ *SeumalT1*Δ
11672	AGGTCCAAGTCCTCTGTAAG	Diagnostic out-out *SeumalT1*Δ
12273	CATGTCGCACAGATTAGAGG	Diagnostic PCR Δ*agt1*
12274	TCGACCAAGAAGGTACTGAG	Diagnostic PCR Δ*agt1*
12917	ATGGTGAAGTTATATAACAAATTGCTCGGCACACTCGCCGTGGGCGTCGGATCTGTCTGGAACGTATTTTAGATTCCTGA	Integration on *PGK1_p_-ScMAL13-TEF2_t_* in *SeSGA1*
12918	TTAAAAGGTGTTTAGAATTTCTTGTCTTATTTGATGGGCGTCCCAAAATGAGGTGTAGGAAGGAAACGTAAATTACAAGG	Integration on *PGK1_p_-ScMAL13-TEF2_t_* in *SeSGA1*
12319	ATGGTGAAGTTATATAACAAATTGCTCGGCACACTCGCCGTGGGCGTCGGATCTGTCTGGGCGTGTGGAAGAACGATTAC	Integration on *TDH_p_-ScMAL12-ADH1_t_* in *SeSGA1*
12320	TTAAAAGGTGTTTAGAATTTCTTGTCTTATTTGATGGGCGTCCCAAAATGAGGTGTAGGAAAGCTGGAGCTCAGTTTATC	Integration on *TDH_p_-ScMAL12-ADH1_t_* in *SeSGA1*
12635	CACGAACCATGTCCGTGTAG	Diagnostic out-out *SeAGT1*
12636	GTTGGACGTTCCGGCATAGC	Diagnostic out-out *SeAGT1*
4224	TTGATGTAAATATCTAGGAAATACACTTG	Diagnostic out-out *ScAGT1*
4226	ACTCGTACAAGGTGCTTTTAACTTG	Diagnostic out-out *ScAGT1*
8570	GCGCTTTACATTCAGATCCCGAG	Diagnostic S. cerevisiae F
8571	TAAGTTGGTTGTCAGCAAGATTG	Diagnostic S. cerevisiae R
8572	GTCCCTGTACCAATTTAATATTGCGC	Diagnostic S. eubayanus F
8573	TTTCACATCTCTTAGTCTTTTCCAGACG	Diagnostic S. eubayanus F
3289	CATACGTTGAAACTACGGCAAAGG	Diagnostic out-out gRNA
7236	CGGTTAGAGCGGATGTGGGG	Diagnostic out-out gRNA
901	CTGCTGTAACCCGTACATGC	Diagnostic out-out gRNA
15866	GGCTTGGAATATTTTGTGCG	Diagnostic out-in MALR02/07/16
15867	CATTGTGATGAGGGTCCTAG	Diagnostic out-in MALR02/07
15868	CTCACCATCTTCGTTTAACATC	Diagnostic out-in MALR16

a “out-out” refers to the position of the primers located outside the open reading frame targeted by the gRNA.

*SeMALS1* and *SeMALS2* were amplified from CDFM21L.1 genomic DNA with Phusion High-Fidelity DNA polymerase (Thermo Scientific), with the primers pairs 14451/14453 and 14452/14453, respectively. Each primer pair carried a 30-bp extension complimentary to the plasmid backbone of pUDE044 (63), which was PCR amplified using Phusion High-Fidelity DNA polymerase (Thermo Scientific) and the primer pair 14449/14450. Resulting amplicons were assembled using NEBuilder HiFi DNA Assembly (New England Biolabs), resulting in plasmids pUDE843 (*SeMALS1*) and pUDE844 (*SeMALS2*) that were verified by Sanger sequencing (Baseclear).

S. cerevisiae *ScMAL13*, the *ScPGK1* promoter (*ScPGK1_p_*), and the *ScTEF2* terminator (*ScTEF2_t_*) were amplified from CEN.PK113-7D genomic DNA with Phusion High-Fidelity DNA polymerase (Thermo Scientific), with the primer pairs 12915/12916, 9421/9422, and 10884/10885, respectively. Fragments were gel purified and used with pYTK074, pYTK082, and pYTK083 in Golden Gate assembly according to the yeast toolkit protocol (64), resulting in pUDE780, which was verified by Sanger sequencing (Baseclear).

Guide RNA (gRNA) sequences for deletion of *SeMALT1* and *SeAGT1* in CDFM21L.1 were designed as described previously (44). The DNA sequences encoding these gRNAs were synthesized at GeneArt (Thermo Scientific) and were delivered in pUD631 and pUD634, respectively. The gRNA spacer sequences (*SeMALT1*, 5′-CCCCGATATTCTTTACACTA-3′; *SeAGT1*, 5′- AGCTTTGCGAAAATATCCAA-3′) and the structural gRNA sequence were flanked at their 5′ ends by a hammerhead ribozyme (HH) and at their 3′ ends by a hepatitis delta virus ribozyme (HDV) (65). The HH-gRNA-HDV fragment was flanked on both ends with a BsaI site for further cloning (44, 66). Plasmids pUDP091 (gRNA*_SeMALT1_*) and pUDP090 (gRNA*_SeAGT1_*) were constructed by Golden Gate cloning by digesting pUDP004 and the gRNA-carrying plasmid (pUD631 and pUD634, respectively) using BsaI and ligating with T4 ligase (67). Correct assembly was verified by restriction analysis with PdmI (Thermo Scientific) and Sanger sequencing (Baseclear).

### Strain construction.

S. cerevisiae IMZ616, which cannot grow on α-glucosides (41), was used as a host to test functionality of individual S. eubayanus (putative) maltose transporter genes (13). S. cerevisiae IMX1702 was constructed by integrating *ScTDH3_p_-ScMAL12-ScADH1_t_* and *ScTEF1_p_-SeMALT1-ScCYC1_t_* at the *ScSGA1* locus of strain IMZ616. A fragment containing the *ScTDH3_p_-ScMAL12-ScADH1_t_* transcriptional unit was PCR amplified using Phusion High-Fidelity DNA polymerase (Thermo Scientific) from pUDE044 with the primer pair 9596/9355, which included a 5′ extension homologous to the upstream region of the *ScSGA1* locus and an extension homologous to the cotransformed transporter fragment, respectively. The DNA fragment carrying the S. eubayanus *SeMALT1* maltose symporter (*ScTEF1_p_-SeMALT1-ScCYC1_t_*) was PCR amplified from pUD794 using the primer pair 9036/9039, which included a 5′ extension homologous to the cotransformed transporter fragment and an extension homologous to the downstream region of the *ScSGA1* locus, respectively. To facilitate integration in strain IMZ616, the two PCR fragments were cotransformed with plasmid pUDR119 (*amdS*), which expressed a gRNA targeting *ScSGA1* (spacer sequence: 5′-ATTGACCACTGGAATTCTTC-3′) (68). The plasmid and repair fragments were transformed using the LiAc yeast transformation protocol (69), and transformed cells were plated on SM_Ace_G. Correct integration was verified by diagnostic PCR with the primers pair 4226/4224. Strains S. cerevisiae IMX1704, IMX1706, and IMX1708 were constructed following the same principle, but instead of using pUD794 to generate the transporter fragment, pUD795, pUD796, and pUD797 were used to PCR amplify *ScTEF1_p_-SeMALT2-ScCYC1_t_*, *ScTEF1_p_-SeMALT3-ScCYC1_t_*, and *ScTEF1_p_-SeAGT1-ScCYC1_t_* respectively. IMX1313 was constructed in a similar way using only *ScTEF1_p_-ScMAL31-ScCYC1_t_* amplified with the primer pair 9036/11018 which contain 5′ and 3′ extensions homologous to the upstream and downstream regions of the *ScSGA1* locus. Correct integration was verified by diagnostic PCR with the primer pair 4226/4224 (see Fig. S3 in the supplemental material). All PCR-amplified genes were Sanger sequenced (BaseClear). IMX1313 was grown on YPD medium to lose pUDR119 (*URA3*) and pUDC156 (*amdS*). An isolate unable to grown on SMG without uracil and with acetamide was selected and named IMX1313Δ. This strain was able to grow on SMG supplemented with 150 mg liter^−1^ uracil.

To assess functionality of CDFM21L.1 *SeMALS1*, IMX1313Δ was transformed with 100 ng of pUDE843 (*ScTDH3_p_-SeMALS1-ScADH1_t_*) by electroporation (44), resulting in strain IMZ752. Transformants were selected on SMG plates after 5 days of incubation at 20°C and validated by PCR (DreamTaq polymerase; Thermo Scientific) using the primer pair 14454/14455 (Fig. S3). Similarly, functionality of the *SeMALS2* maltase gene of CDFM21L.1 was assessed by transforming IMX1313Δ with pUDE844 (*ScTDH3_p_-SeMALS2-ScADH1_t_*), resulting in strain IMZ753.

S. eubayanus IMK820 (*SemalT1Δ*) was constructed by transforming CDFM21L.1 with 200 ng of pUDP091 and 1 μg of a 120-bp repair fragment obtained by mixing an equimolar amount of primers 12442/12443, as previously described (44). As a control, the same transformation was performed without including the repair DNA fragment. Transformants were selected on SM_Ace_G plates. S. eubayanus IMK823 (*Seagt1Δ*) was constructed similarly, using pUDP090 and the primer pair 11320/11321. Deletion of *SemalT1* was verified by PCR with the primer pair 11671/11672 and Sanger sequencing. The *Seagt1* deletion was verified by PCR using the primer pair 12273/12274 and by Illumina whole-genome sequencing and read alignment to the reference genome of CDFM21L.1.

Strains IMX1765, IMX1939, and IMX1940 were constructed by inserting *ScPGK1_p_-ScMAL13-ScTEF2_t_* at the *SeSGA1* locus of CDFM21L.1, IMK820, and IMK823, respectively. A repair fragment containing *ScPGK1_p_-ScMAL13-ScTEF2_t_* was amplified from pUDE780 with the primer pair 12917/12918. Strains CDFM21L.1, IMK820, and IMK823 were transformed by electroporation by addition of 350 ng of repair fragment and 560 ng of pUDP052 (*amdS*) into the cells as previously described (44). Transformants were plated on SM_Ace_G and incubated at 20°C. IMX1762 was constructed similarly using a repair fragment with *ScTDH3_p_-ScMAL12-ScADH1_t_* amplified from pUDE044 with the primer pair 12319/12320. Strains were verified by PCR using the primer pair 12635/12636 and Sanger sequencing.

### Hybrid construction.

The S. cerevisiae × S. eubayanus hybrid HTSH020 was constructed by spore-to-spore mating. The S. eubayanus strain CDFM21L.1 and the S. cerevisiae strain CBC-1 were grown in 20 ml of YPD medium at 20°C until late exponential phase. Cells were centrifuged for 5 min at 1,000 × *g* and washed twice in demineralized water. Cells were resuspended in 20 ml of sporulation medium and incubated for 64 h at 20°C. Presence of spores was verified by microscopy. Asci were harvested by centrifugation for 5 min at 1,000 × *g*, washed with demineralized water, resuspended in 100 μl of demineralized water containing 100 U/ml of Zymolyase (MP Bio, Santa Ana, CA), and incubated for 10 min at 30°C. Spores were washed and plated on the edge of a YPD agar plate. Spores from the two strains were brought in contact with each other with an MSM System 400 micromanipulator (Singer Instruments, Watchet, United Kingdom). Zygote formation was observed after 6 to 8 h. Emerging colonies were restreaked twice on SM with 2% maltose at 12°C. Successful hybridization was verified by multiplex PCR using DreamTaq DNA polymerase (Thermo Scientific) by amplifying the S. cerevisiae-specific *MEX67* gene with the primer pair 8570/8571 and by amplifying the S. eubayanus-specific gene *SeFSY1* with the primer pair 8572/8573 (Fig. S4), as previously described (70).

### Illumina sequencing.

Genomic DNA of S. eubayanus strains CDFM21L.1 and ABFM5L.1, S. cerevisiae strain CBC-1, and S. cerevisiae × S. eubayanus strain HTSH020 was isolated as previously described (4). Paired-end sequencing (2 by 150 bp) was performed on a 350-bp PCR-free insert library using a HiSeq 2500 system (Illumina, San Diego, CA) by Novogene (HK) Company, Ltd. (Hong Kong, China). Genomic DNA of the strains CBC-1 and HTSH020 was sequenced in-house on a MiSeq sequencer (Illumina) with 300-bp paired-end reads using a PCR-free library preparation.

### MinION long-read sequencing.

For long-read sequencing, a one-dimensional (1D) sequencing library (SQK-LSK108) was prepared for CDFM21L.1 and CBC-1 and loaded onto an FLO-MIN106 (R9.4) flow cell, connected to a MinION Mk1B unit (Oxford Nanopore Technology, Oxford, United Kingdom), according to the manufacturer’s instructions. MinKNOW software (version 1.5.12; Oxford Nanopore Technology) was used for quality control of active pores and for sequencing. Raw files generated by MinKNOW were base called using Albacore (version 1.1.0; Oxford Nanopore Technology). Reads with a minimum length of 1,000 bp were extracted in fastq format. For CDFM21L.1, 841.6 Mb of sequence with an average read length of 4.83 kb was obtained, and for CBC-1 3.04 Gb of sequence with an average read length of 7.27 kb was obtained.

### *De novo* assembly.

*De novo* assembly of the Oxford Nanopore MinION data set was performed using Canu (version 1.4; setting: genomesize = 12m) (71). Assembly correctness was assessed using Pilon (72) and further corrected by polishing of sequencing/assembly errors by aligning Illumina reads with the Burrows-Wheeler Aligner (BWA) (73) using correction of only single nucleotide polymorphisms (SNPs) and short indels (–fix bases parameter). For HTSH020, an artificial reference genome was made by combining the assembly of CBC-1 and CDFM21L.1. The genome assemblies were annotated using the MAKER2 annotation pipeline (version 2.31.9) (74), using SNAP (version 2013-11-29) (75) and Augustus (version 3.2.3) (76) as *ab initio* gene predictors. S. cerevisiae S288C expressed sequence tag (EST) and protein sequences were obtained from the *Saccharomyces* Genome Database (SGD [http://www.yeastgenome.org/]) and were aligned using BLASTX on the obtained polished sequence assembly (BLAST, version 2.2.28+) (77). Predicted translated protein sequences of the final gene model were aligned to the S. cerevisiae S288C protein Swiss-Prot database using BLASTP (http://www.uniprot.org/). Custom-made Perl scripts were used to map systematic names to the annotated gene names (Table S1). Error rates in nanopore sequencing data were estimated from the *q* score (Phred scaled) per read, as calculated by the base caller Albacore (version 1.1.0) (Oxford Nanopore Technology). Average *q* score was used to calculate the error *P* = 10^q/10^.

### RNA isolation.

CDFM21L.1, IMX1765, IMX1939, and HTSH020 were grown in SMG, SMM, and SMMt until mid-exponential phase (OD_660_ of 12 for SMG/SMM and of OD_660_ 15 for SMMt). Culture samples corresponding to ca. 200 mg of biomass dry weight were directly quenched in liquid nitrogen. The samples were processed, and total RNA was extracted as previously described (78). Prior to cDNA synthesis, purity, concentration, and integrity of the RNA in the samples was assessed with Nanodrop (Thermo Scientific), Qubit (Thermo Scientific), and Tapestation 220 with RNA Screen Tape (Agilent Technologies), respectively, according the manufacturers’ recommendations. cDNA libraries were prepared using a TruSeq RNA version 2 kit (Illumina). Paired-end sequencing (2 by 150 bp) was performed on a 300-bp PCR-free insert library on a HiSeq 2500 system (Illumina) at Novogene (HK) Company, Ltd. (Hong Kong, China). Duplicate biological samples were processed, generating an average sequence quantity of 23.7 million reads per sample. Reads were aligned to the CDFM21L.1 reference assembly (GEO [https://www.ncbi.nlm.nih.gov/geo/]) using a two-pass STAR (79) procedure. In the first pass, splice junctions were assembled and used to inform the second round of alignments. Introns between 15 and 4,000 bp were allowed, and soft clipping was disabled to prevent low-quality reads from being spuriously aligned. Ambiguously mapped reads were removed from the data set. Expression levels for each transcript were quantified using htseq-count (80) in union mode. For the gene of interest, the number of fragments per kilo base per million reads (FPKM) mapped was calculated by applying the fpkm method from the edgeR package (81, 82). Differential expression analysis was performed using DESeq (83).

### Data availability.

The sequencing data were deposited at NCBI (https://www.ncbi.nlm.nih.gov/) under BioProject accession number PRJNA528469, and the transcriptomics data were deposited in the Gene Expression Omnibus (GEO) database (https://www.ncbi.nlm.nih.gov/geo/) under accession number GSE133146.

## Supplementary Material

Supplemental file 1

Supplemental file 2

Supplemental file 3
