# Supplementary material for: Himalayan Saccharomyces eubayanus Genome Sequences Reveal Genetic Markers Explaining Heterotic Maltotriose Consumption by Saccharomyces pastorianus Hybrids
Source: Appl Environ Microbiol. 2019 Oct 30;85(22):e01516-19. doi: 10.1128/AEM.01516-19 (PMC6821976; doi:10.1128/AEM.01516-19)
Supplement: Supplemental file 1 [file AEM.01516-19-s0001.pdf]

Supplementary Tables and Figures

The genome sequences of Himalayan *Saccharomyces eubayanus* revealed genetic markers explaining heterotic maltotriose consumption by hybrid *Saccharomyces pastorianus*.

Heterotic origin of maltotriose consumption in *Saccharomyces pastorianus*

Nick Brouwers[a] , Anja Brickwedde[a], Arthur R. Gorter de Vries[a], Marcel van den Broek[a], Susan M. Weening[a], Lieke van den Eijnden[a], Jasper A. Diderich[a], Feng-Yan Bai[b], Jack T. Pronk[a] and Jean-Marc G. Daran[a*]

[a]Department of Biotechnology, Delft University of Technology, Van der Maasweg 9, 2629 HZ Delft, The Netherlands.

[b]State Key Laboratory of Mycology, Institute of Microbiology, Chinese Academy of Sciences, Beijing, 100101, China.

* Corresponding author: Jean-Marc G. Daran, j.g.daran@tudelft.nl, Department of Biotechnology, Delft University of Technology, van der Maasweg 9, 2629 HZ Delft, The Netherlands

Supplementary Table 2: Statistical significance assessment ($^{student\ t\text{-}test}p_{value}$)of Expression levels of maltose metabolism genes in *S. eubayanus* IMX1765 (MATa/MATα Sesga1Δ::ScMAL13 / Sesga1Δ::ScMAL13) mid-exponential phase grown cells on glucose, maltose and maltotriose (Figure 6). p-values were determined using Student's t-test.

$^{t\text{-}test}p_{value}$	Glc vs Mal	Glc vs Mtt	Mal vs Mtt
SeMALT1	2.85E-03	5.18E-04	1.11E-02
SeMALT2	7.49E-02	4.01E-02	2.27E-01
SeMALT3	3.38E-05	7.22E-03	1.29E-01
SeAGT1	1.54E-04	4.91E-04	3.23E-01
SeMALS1	1.68E-03	6.37E-04	5.47E-03
SeMALS2	1.75E-03	3.42E-03	5.25E-03
SeMALR1	2.90E-02	1.92E-02	2.17E-01
SeMALR2	5.90E-03	8.17E-04	3.64E-02
SeMALR3	2.81E-03	3.36E-03	1.54E-01
ScMAL13	8.24E-01	3.94E-01	7.53E-01

Supplementary Table 3: Statistical significance assessment ($^{student\ t\text{-}test}p_{value}$) of expression levels of maltose metabolism genes in interspecific hybrid (Sc X Se) HTS020 mid-exponential phase grown cells on glucose, maltose and maltotriose (Figure 7). p-values were determined using Student's t-test.

	Glc vs Mal	Glc vs Mtt	Mal vs Mtt
ScMALR3	4.62E-01	1.35E-01	9.40E-02
ScMALR2	1.20E-02	5.14E-02	2.36E-03
ScMALR1	2.05E-03	2.32E-03	1.66E-01
SeMALR3	3.87E-04	2.00E-04	1.78E-03
SeMALR2	1.35E-01	1.61E-02	2.12E-01
SeMALR1	3.86E-03	4.39E-03	3.86E-02
ScMALS2	6.35E-05	1.06E-03	9.49E-05
ScMALS1	5.35E-05	9.07E-06	7.02E-05
SeMALS2	2.90E-04	5.83E-05	3.16E-04
SeMALS1	3.54E-03	2.87E-03	4.11E-03
ScMALT2	4.28E-05	7.26E-04	1.61E-04
ScMALT1	7.62E-03	4.99E-03	1.60E-02
SeMALT3	9.71E-04	2.60E-02	1.78E-03
SeMALT2	6.23E-04	8.96E-02	8.95E-04
SeMALT1	8.02E-04	2.56E-03	3.29E-03
SeAGT1	4.73E-03	4.23E-03	6.65E-03

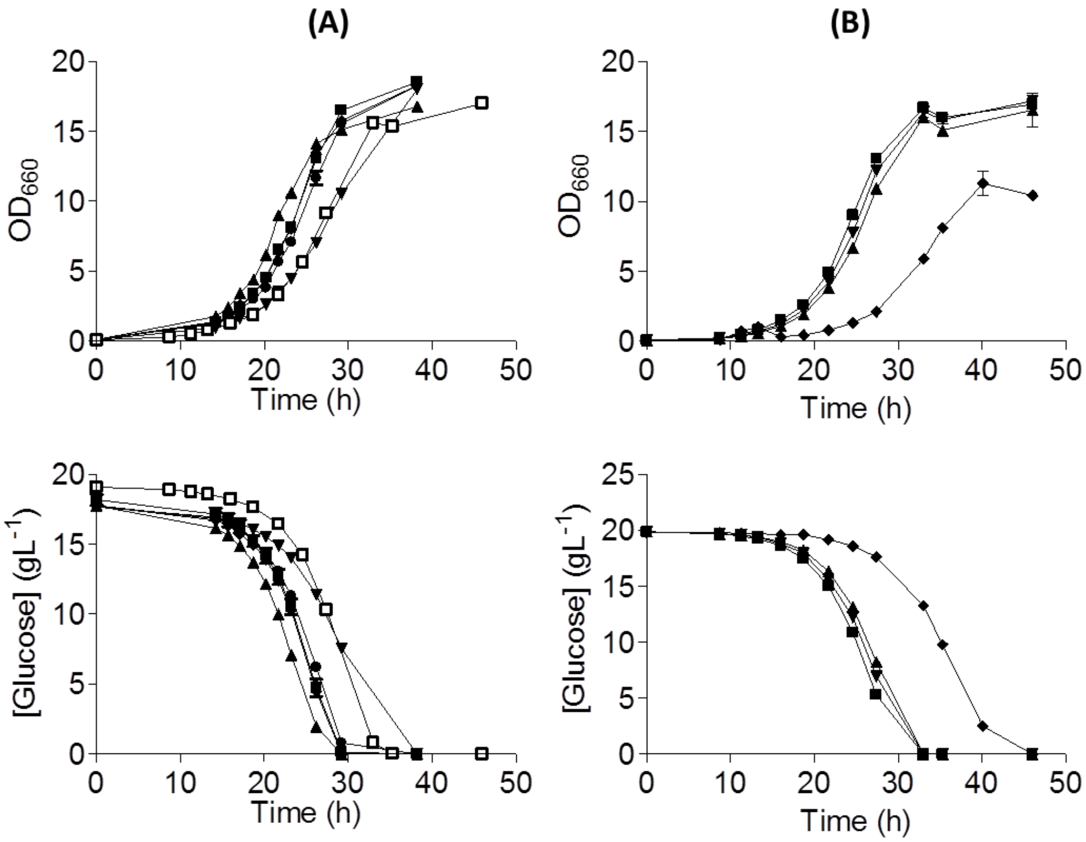

Supplementary Figure 1 Overexpressing and knockout strains grown on SM glucose. (A) IMZ616 (■), IMX1365 overexpressing *ScMAL11* (▲), IMX1702 overexpressing *SeMALT1* (▼), IMX1704 overexpressing *SeMALT2* (◆) IMX 1706 overexpressing *SeMALT3* (●) and IMX 1708 overexpressing SeAGT1 (□) were grown on SM 2% glucose at 20 °C. Growth was monitored based on optical density (OD_{660nm}) and glucose concentration in culture supernatant was measured by HPLC. Data are presented as average and standard deviation of two biological replicates. **(B)** *S. eubayanus* strains IMK820 (■), IMK823 (▲), IMX1939 (▼) and IMX1940 (◆) were characterized on SM glucose at 20 °C. Growth was monitored based on optical density (OD_{660nm}) and glucose concentration in culture supernatant was measured by HPLC. Data are presented as average and standard deviation of two biological replicates.

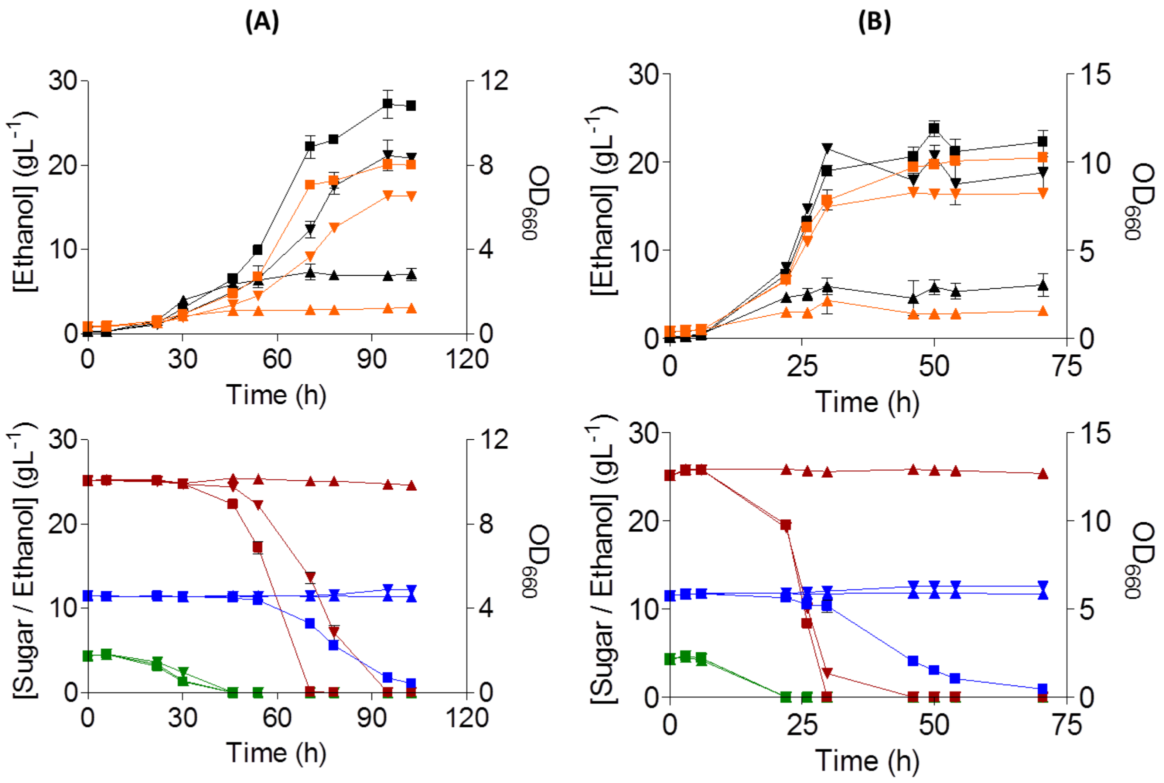

Supplementary Figure 2 Hybridization of maltotriose deficient *S. cerevisiae* and *S. eubayanus* leading to crosstalk restoring maltotriose utilization, explains *S. pastorianus* phenotype. Characterization of *S. cerevisiae* CBC-1 (▼), *S.* eubayanus CDFM21L.1 (▲) and hybrid HTS020 (■) on mock wort at 12 °C **(A)** and 20 °C **(B)**. Growth was monitored based on OD_{660nm} (black). Consumption of glucose (green), maltose (red), maltotriose (blue) and production of ethanol (orange) was measured from supernatant by HPLC. Data represents average and standard deviation from biological triplicates.

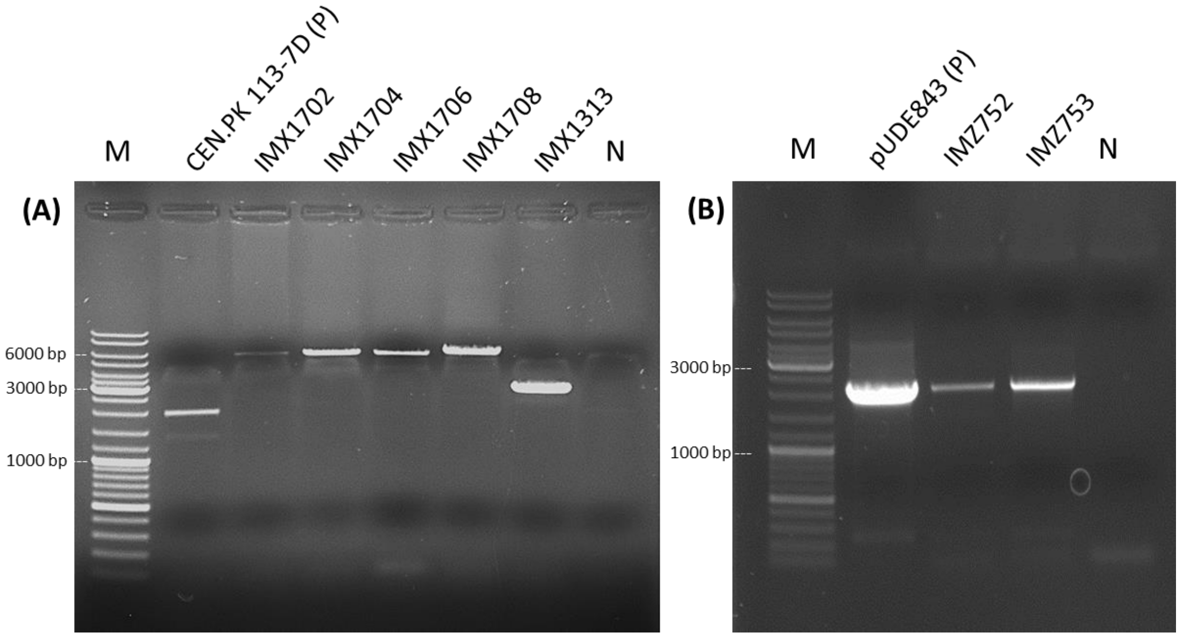

Supplementary Figure 3 Verification PCR of IMX1702-IMX1708, IMZ752 and IMZ753. (A) Outside-outside insert PCR amplification of the SGA1 locus or integrated fragments was done using primer pair 4226/4224 . As positive control CEN.PK 113-7D was used. **(B)** Colony PCR to verify the presence of pUDE843 and pUDE844 in IMX1313Δ with primer pair 14454/14455. Gels (TAE 1% agarose) were run at 100V for25 minutes. As marker (M) GeneRuler DNA Ladder Mix (Thermo Scientific) was used.

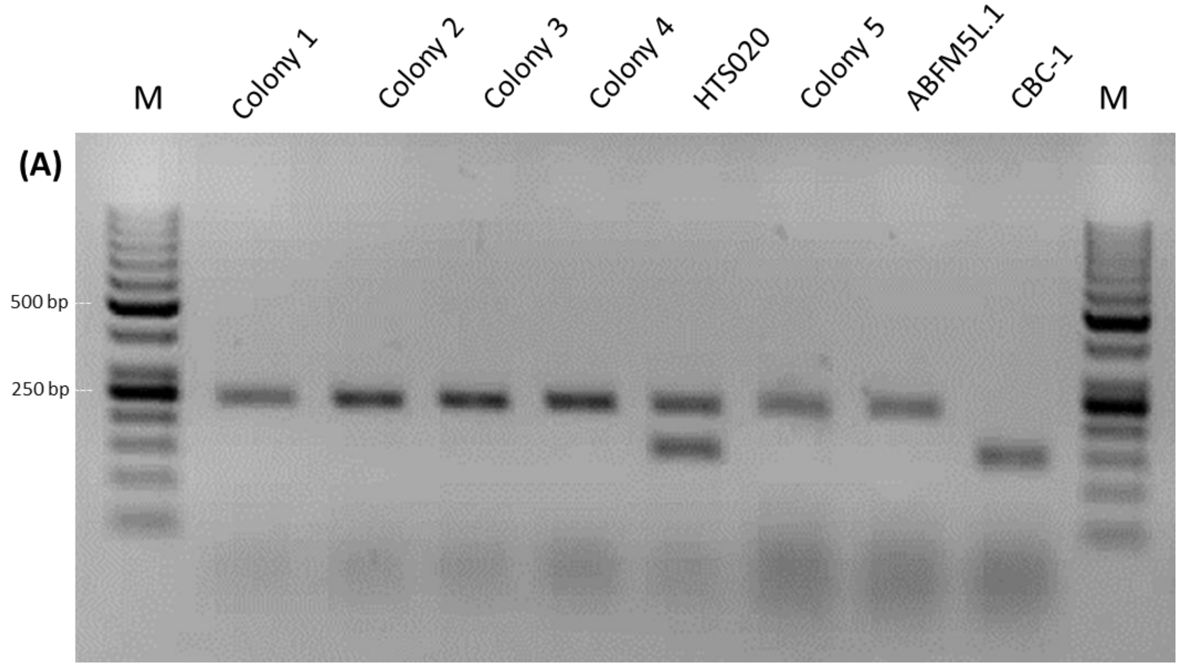

Supplementary Figure 4 Verification of successful hybridization by multiplex PCR. Multiplex PCR using primer pairs 8570/8571 (*S. cerevisiae* specific) and 8572/8573 (*S. eubayanus* specific). Lanes contain single colony isolates and ABFM5L.1 and CBC-1 as controls. Gels (TBE 2% agarose) were run at 120V for 40 minutes. As marker (M) GeneRuler 50 bp DNA Ladder (Thermo Scientific) was used.
